# Protein kinase CK2α′ as a dual modulator of neuroimmune signaling and synaptic dysfunction in tauopathy

**DOI:** 10.1186/s40035-026-00563-3

**Published:** 2026-07-09

**Authors:** Angel White, Peter Gavrilyuk, Persephone Gu, Rafael Falcon-Moya, Reid Thurston, Amal Fickak, Nicholas B. Rozema, Prarthana Keshavaram, Scott Vermilyea, Riley Schlichte, Joyce Meints, Ying Zhang, Alfonso Araque, Michael K. Lee, Rocio Gomez-Pastor

**Affiliations:** 1 School of Medicine, Department of Neuroscience, University of Minnesota, Minneapolis, MN USA; 2 Minnesota Supercomputing Institute, University of Minnesota, Minneapolis, MN USA

**Keywords:** Tauopathy, CK2, CK2α′, Microglia, Phagocytosis, Synaptic function

## Abstract

**Background:**

Tauopathies are a group of neurodegenerative diseases characterized by tau accumulation, neuroinflammation, and synaptic dysfunction, yet effective treatments remain elusive. Protein kinase CK2 is a holoenzyme composed of two regulatory (CK2β) and two catalytic subunits (CK2α and CK2α′) and has been linked to multiple aspects of tau pathology. However, genetic evidence defining the specific contributions of CK2 subunits to tau phosphorylation and tauopathy remains lacking. Elucidating subunit-specific roles is critical for the rational development of CK2-targeted therapies.

**Methods:**

To investigate the impact of CK2 in tauopathy, Neuro-2a and primary cell cultures expressing mutant tau were treated with siRNAs targeting the two catalytic subunits of CK2, CK2α and CK2α′. In addition, the PS19 mouse model of tauopathy was bred to be haploinsufficient for the catalytic subunit CK2α′. Changes in pathology and symptomatology were analyzed via immunohistochemistry, immunoblotting, RNA-sequencing, in situ hybridization, electrophysiology, and Barnes Maze.

**Results:**

We found that the expression of the catalytic subunit CK2α′, but not catalytic CK2α or regulatory CK2β subunits, was elevated in postmortem brains of dementia patients and in the hippocampus of PS19 tauopathy mice, especially in neurons and microglia. Using a haploinsufficient model of CK2α′ in PS19 mice, we demonstrated that the PS19:CK2α′^(+/−)^ mice had significantly decreased phosphorylated tau and total tau burden in the hippocampus and cortex. CK2α′ depletion also attenuated microglial activation, pro-inflammatory cytokine production and microglia synaptic engulfment, and enhanced synaptic gene expression, synaptic density, and long-term potentiation. Importantly, CK2α′ haploinsufficiency rescued cognitive deficits assessed in the Barnes maze.

**Conclusions:**

Here, we show CK2α′, one of the two catalytic subunits of CK2, as a novel regulator of tau-mediated neurodegeneration. These effects appear to be mediated through both neuronal and glial functions and may involve CK2α′-dependent modulation of tau phosphorylation as well as neuroinflammatory and immune signaling pathways. These findings identify CK2α′ as a mechanistically defined and potentially druggable target for therapeutic strategies aimed at modifying tau-driven neurodegeneration.

**Supplementary Information:**

The online version contains supplementary material available at 10.1186/s40035-026-00563-3.

## Background

Tauopathies encompass a broad range of neurodegenerative conditions, most notably Alzheimer’s disease (AD) and related dementias (ADRD), for which no cure currently exists [1–3]. Tauopathies are typically marked by progressive neuronal loss, synaptic and cognitive dysfunction, and widespread inflammation in both the central nervous system and peripheral tissues [4–8]. A deeper understanding of the molecular mechanisms underlying tauopathies and their downstream consequences is essential for developing effective disease-modifying therapies. One key area of research is the role neuroinflammation plays in the progression of tauopathies. These disorders are characterized by chronic, excessive inflammatory responses to abnormal tau proteins [5, 6, 9]. This persistent inflammation can exacerbate tau pathology by promoting sustained neuronal damage and dysfunction [9–12]. However, despite ongoing research, the mechanisms that connect neuroinflammation and tau pathology are still poorly understood.

Protein kinase CK2 (formerly known as Casein Kinase II) is a serine/threonine kinase that has emerged as an important regulator of neuroinflammation and protein homeostasis in several neurodegenerative diseases [13–15], and has previously been connected to tau pathology in AD [16–18]. However, its specific role in the inflammatory pathways associated with tauopathies remains poorly defined. Interestingly, CK2 has demonstrated potential as a therapeutic target in various neurodegenerative contexts [14, 15]. In AD, CK2 has been implicated in processes such as synaptic plasticity [19, 20], amyloid precursor protein (APP) processing [21–23], and tau accumulation [16, 24, 25], highlighting its relevance for further investigation.

CK2 is a tetrameric enzyme composed of 2 regulatory β subunits (CK2β) and 2 catalytic subunits α (CK2α) or α′ (CK2α′) [13]. CK2α and CK2α′ are highly similar in structure but display differential expression and substrate specificity [26–32]. CK2α is ubiquitously expressed across tissues and has hundreds of substrates, while CK2α′ expression is more restricted, with highest levels in the testes and brain, and has fewer validated substrates [26–32]. Another important difference between these two catalytic subunits is the embryonic lethality of CK2α knock-out mice, while complete deletion of CK2α′ does not have any major defects other than male sterility [31, 32]. Recent studies show that CK2α′ is selectively upregulated in Huntington’s disease (HD), and partial genetic reduction of CK2α′ in HD mouse models improves synaptic function, reduces protein aggregation, and alleviates behavioral deficits, indicating its key role in disease pathology [14, 15].

CK2 was one of the first protein kinases identified to be altered in an AD brain [33, 34], but its role in AD has been somewhat controversial due to discrepancies in the ability to detect consistent alterations in different affected tissues. Early studies using pan-CK2 antibodies (non-selective for CK2α′/CK2α subunits) showed increased levels of total CK2 in the hippocampus and frontal cortex of AD mouse models and patients with AD, especially in glial cells [35], but others have reported opposite results [33, 36, 37]. In those studies where levels of CK2 were elevated, the authors associated the increase in CK2 immunoreactivity with the activation of neuroinflammatory processes and AD pathology [35].

Although genetic studies have not yet clarified whether CK2α and CK2α′ differentially contribute to AD pathology, multiple lines of evidence implicate CK2 in tau phosphorylation and tau-mediated dysfunction. Kinase inhibitor screens identified CK2 as a major driver of okadaic-acid–induced phosphorylated tau (pTau) in N2a cells [17]. CK2-mediated phosphorylation of the PP2A inhibitor SET increases pTau levels in neurons exposed to Aβ or overexpressing hTau [18]. Moreover, mice overexpressing CK2 exhibit elevated pTau, impaired synaptic plasticity, and cognitive deficits reminiscent of tauopathies [18]. Despite this, most studies have treated CK2 as a single holoenzyme, overlooking the known differences in substrate specificity between CK2α and CK2α′. Determining whether these catalytic subunits play distinct roles in tauopathy is essential for developing targeted therapies, especially given the current lack of subunit-selective CK2 inhibitors and the opportunity this presents for designing more precise and effective treatments.

Given the emerging links between CK2α′ dysregulation and tau phosphorylation in neurodegeneration, this study aimed to define the role of CK2α′ in tau pathology, neuroinflammation, synaptic dysfunction, and cognitive deficits associated with tauopathies, while evaluating its potential as a therapeutic target.

## Materials and methods

### Study design

In this study we report the upregulation of CK2α′ in AD human samples and mouse models and investigated the role of CK2α′ in tauopathy. We hypothesized that CK2α′ contributes to tau-associated pathology and that reducing its levels would improve outcomes in tauopathy models. Data were collected from AD/ADRD post-mortem human tissues as well as AD mouse and cell models. Methods used in this study include Western blotting, immunostaining (tissue and cells), bulk RNA-Seq, electrophysiology and behavioral assays. Publicly available human genomic datasets were also analyzed, as described.

For mouse experiments, animals were assigned to cohorts at birth based on genotype and aged to predetermined time points; no additional randomization was performed. Sample sizes for all experiments were chosen based on previous literature and our prior experience with similar experiments; no formal power calculations were performed, and exact sample sizes are detailed in figure legends. Equal numbers of male and female mice were used. Data are presented as mouse averages unless otherwise detailed in the figure legend.

Experimenters were not blinded during behavioral testing. However, all image analysis was performed on blinded samples to reduce experimenter bias.

### Cell culture: culturing, transfections, and immunocytochemistry

Neuro-2a cells (N2a; ATCC CCL-131) were cultured in DMEM with 10% fetal bovine serum and penicillin/streptomycin at 37 °C and 5% CO₂. For silencing experiments, cells were plated in 12-well plates or Matrigel-coated coverslips, and transfected with siRNA targeting CK2α (siCK2α) or CK2α′ (siCK2α′) (Qiagen, Hilden, Germnay) or scrambled RNA (ScrRNA) using DharmaFECT 1 (25 nM) (Horizon Discovery, Lafayette, CO). After 24 h, cells were transfected with pEGFP-C1 or Tau-P301L-EGFP using jetOptimus (Polyplus-transfection, Illkirchm, France) and processed 24 h later. For immunocytochemistry, cells were fixed, blocked in 5% normal goat serum (NGS) in TBST, incubated overnight with AT8 primary antibody (Invitrogen/Thermo Fisher Scientific, Waltham, MA), followed by Alexa-fluor secondary antibodies (Invitrogen/Thermo Fisher Scientific), and mounted with ProLong Gold for imaging.

### Primary cell culture

Primary cell culture was performed as previously described [38]. Briefly the hippocampus was dissected from mouse pups between postnatal day 0–2, placed in cold Hibernate-A medium (BrainBits LLC, Springfield, IL), then transferred to digestion medium containing Hibernate-A-CaCl_2_ (BrainBits, Cordova, TN), papain, *L*-Cysteine, and EDTA for 15 min at 37 °C with occasional gentle shaking. The hippocampi were then washed in a digestion inhibitor solution before being triturated with a pasteur pipette. Cells were plated in a 35-mm cell culture dish at a density of 1 × 10^6^ cells/plate and placed in a 37 °C/5% CO_2_ incubator. The next day the medium was replaced with NbActiv4 (BrainBits, Cordova, TN) +FdU mitotic inhibitor.

For RNA extraction, cells were transfected at 14 days in vitro (DIV) with siCK2α′ or ScrRNA as described in cell culturing techniques, and RNA was extracted 48 h later as described. For spine imaging, cells were transfected with a plasmid expressing DsRed (Clonetech; Mountain View, CA) and Tau-P301S-EGFP under the CMV promotor in the pRK5 vector (a gift from the laboratory of Dr. Michael Lee, University of Minnesota, Minneapolis, MN) at 9 DIV using the LTX DNA transfection kit (#15338030, Thermo Fisher Scientific, Waltham, MA) and then transfected with siCK2α′ or ScrRNA at 21 DIV. Forty-eight hours after siRNA transfection (23 DIV), cells were live imaged using a Leica Stellaris 8 confocal microscope (Lecia Microsystems) with a gas- and temperature-regulated culture dish chamber. Z-stacks were acquired using a 63× objective with 2.5× digital zoom. Leica software "Lightening" noise reduction was applied to the z-stack series and then maximum projection images were obtained for subsequent analysis.

Dendrite density analysis was performed by an experimenter blinded to the experimental groups. For each image, spines were counted in two different 20-μm long dendrite regions. Dendritic density was then calculated and averaged per image and normalized to the control.

### Mouse lines

For this study we used the B6;C3-Tg(Prnp-MPAT-P301S)PS19Vle/J (or PS19) [39] mouse line (Jackson, #008169), maintained with purchased B6C3H breeders (obtained from Dr. Karen Ashe at University of Minnesota), and CK2α′^(+/−)^ mice, originally obtained from Dr. Seldin at Boston University (Taconic Biosciences, TF3062) [32] and maintained on the C57Bl/6 J background for several generations. The PS19 and CK2α′^(+/−)^ mice were crossed, generating 4 genotypes of interest, WT (PS19^0/0^;CK2α′^(+/+)^), CK2α′^(+/−)^ (PS19^0/0^;CK2α′^(+/−)^), PS19 (PS19^Tg/0^;CK2α′^(+/+)^), and PS19;CK2α′^(+/−)^ (PS19^Tg/0^;CK2α′^(+/−)^) mice. For all experiments, littermate WT and CK2α′^(+/−)^ controls were used. Two time points were used in experiments: pre-symptomatic (6–7 months) and symptomatic (10–12 months). All mice were housed under standard SPF conditions. All animal care and sacrifice procedures were approved by the University of Minnesota Institutional Animal Care and Use Committee (IACUC) in compliance with the National Institutes of Health guidelines for the care and use of laboratory animals under the approved animal protocol 2307-A41243.

### Human postmortem tissue

Postmortem human frontal cortex (Brodmann’s area 46) tissues from frontotemporal dementia (FTD) patients and control subjects were provided by NeuroBioBank of National Institutes of Health. A total of 6 female (3 control/3 FTD) and 8 male (4 control/4 FTD) samples were used, and samples were age- and sex-matched. Ages ranged 60–77 (average age, 70). More detailed information of specific samples can be found in Additional File 2.

### Immunoblotting

Protein was extracted as previously described [14]. Tissue was homogenized in lysis buffer containing 25 mM Tris–HCl pH 7.4, 150 mM NaCl, 1 mM EDTA, 0.1% SDS, and 1% Triton X-100, incubated on ice for 15–30 min, then SDS was added to a final concentration of 2% and samples were heated at 100 °C for 5 min. Lysates were centrifuged at 13,000× *g* for 20 min at 4 °C and supernatants collected. Protein concentration was determined by BCA assay (Pierce, Thermo Fisher).

Protein samples were separated on 4%–20% SDS Criterion TGX Stain-Free gels (BioRad, Hercules, CA) and transferred to a 0.2-μm nitrocellulose membrane in Tris–Glycine Buffer (25 nM Tris-Base, 200 mM Glycine) at 25 V for 30 min (Trans Turbo Transfer system). Membranes were blocked in 5% milk in TBST (0.5% Tween-20) for 1 h, incubated overnight at 4 °C with primary antibody diluted in 2.5% milk in TBST, then incubated with HRP-conjugated secondary antibody (Cytiva, Marlborough, MA; Amersham 1:5000) for 1 h. Bands were visualized with SuperSignal Chemiluminiscent substrate (Thermo Fisher Scientific) using a GE ImageQuant Las4000 imager. Primary antibodies were rabbit anti-CK2α′ (1:2000; Novus NB100-379), rabbit anti-CK2α (1:1000; Abcam, Cambridge, UK; ab76040), rat anti-Clec7a (1:500; invivoGen, San Diego, CA mabg-mdect), mouse anti-AT8 (1:1000; Invitrogen/Thermo Fisher Scientific, MN1020), mouse anti-At100 (1:1000; Invitrogen/Thermo Fisher Scientific; MN1060), mouse anti-PHF1 (1:1000; a gift from Dr. Michael Lee, Minneapolis, MN; RRID: AB_2315150), mouse anti-Tau-5 (1:1000; Abcam, ab80579), mouse anti-Actin (1:500; Santa Cruz Biotechnology, Dallas, TX, sc-8432), and mouse anti-GAPDH (1:5000; Santa Cruz Biotechnology, sc-365062).

### Cytokine proteome profiling

Cytokine and chemokine levels were assessed using the Proteome Profiler Mouse Cytokine Array Panel (ARY006, R&D Systems, Minneapolis, MN) following manufacturer’s instructions. Frozen hippocampi from 9- to 10-month-old PS19 and PS19;CK2α′^(+/−)^ mice (*n* = 4/genotype; 2F/2M) were homogenized in PBS containing Halt protease inhibitor cocktail/phosphatase inhibitors (Fisher Scientific) and 1% Triton X-100 (Sigma-Aldrich, St. Louis, MO). Lysates were frozen at −80 °C for 15 min, thawed and centrifuged at 10,000× *g* for 5 min. 300 μg of protein were applied per membrane. Imaging was conducted as described for immunoblotting. Spot intensity was quantified using FIJI software and a batch correction was applied. Membrane spots corresponding to 40 cytokines or chemokines were measured according to the manufacturer’s protocol.

### Immunohistochemistry

Immunohistochemistry was conducted as previously described [14, 40, 41] using fluorescent or 3,3′-diaminobenzidine (DAB) detection. Mice were anesthetized (Avertin, 250 mg/kg) and perfused intracardially with Tris-buffered saline (25 mM Tris-base, 135 mM NaCl, 3 mM KCl, pH 7.6) supplemented with 7.5 mM heparin. Brains were fixed with 4% paraformaldehyde at 4 °C for 4–5 days, cryoprotected with 30% sucrose for 4–5 days and embedded in a 2:1 mixture of 30% sucrose in Tris-buffered saline:OCT (Tissue-Tek; Sakura Finetek USA, Torrance, CA). Coronal sections (16 μm) were stored in a 50% glycerol–50% sucrose at −20 °C. Three hippocampal sections were analyzed per mouse.

For immunofluorescent staining, sections were blocked in 5% NGS in TBST for 1 h at room temperature. Primary antibodies were incubated overnight at 4 °C in TBST containing 5% NGS. Secondary Alexa-fluorophore-conjugated antibodies (Invitrogen/Thermo Fisher Scientific) were added (1:200 in TBST with 5% NGS) for 1 h at room temperature. Slides were mounted in ProLong Gold Antifade with DAPI (Invitrogen/Thermo Fisher Scientific) and subsequently imaged. Primary antibodies used and dilutions are as follows: chicken anti-GFAP (1:2000; Millipore Sigma, Burlington, MA; AB5541), rabbit anti-Iba1 (1:500; Fujifilm Wako, Richmond, VA; 019-19741), goat anti-Iba1 (1:500, Fujifilm Wako, 011-27991), mouse anti-NeuN (1:1000; MilliporeSigma, Burlington, MA; MAB377), rat anti-Clec7a (1:1000; invivoGen, mabg-mdect), rabbit anti-PSD-95 (1:500; Invitrogen/Thermo Fisher Scientific; 51-6900) and guinea pig anti-vesicular glutamate transporter 1 (VGlut1) (1:500; MilliporeSigma, AB5905). Imaging of immunofluorescent slices was conducted using Leica Stellaris 8 confocal microscope (Lecia Microsystems, Wetzlar, Germany). For NeuN, Iba1, and GFAP imaging, 20× tiles were acquired with a 16-μm z-dimension and 1-μm step size. For PSD-95 and VGlut1, images were acquired as described for synapse analysis.

For DAB staining, antigen retrieval was performed using either Tris–EDTA pH 9.0 (Biolegend, San Diego, CA; 422703) or Rodent Decloaker (Biocare Medical, Pacheco, CA; RD913) at 80 ºC for 30 min. Sections were blocked at room temperature in 10% NGS/TBST for 1 h, then incubated with primary antibodies overnight at 4 °C in 5% NGS/TBST. Sections were incubated with biotin-conjugated secondaries (Jackson ImmunoResearch Labs, West Grove, PA; Biotin-SP (long spacer) AffiniPure) in 5% NGS/TBST for 1 h, followed by 3% hydrogen peroxide for 20 min. Sections were incubated with tertiary antibodies (VECTASTAIN Elite ABC-HRPS kit, Vector Laboratories, Newark, CA) in 5% NGS/TBST for 1 h per manufacturer’s instructions. DAB chromogen diluted in DAB substrate buffer (Biolegend) was applied for sufficient staining. Sections were counterstained with 0.1% cresly violet, dehydrated, cleared in ethanol/xylene and mounted in Permount mounting medium (Fisher Scientific). Primary antibodies used and dilutions are as follows: mouse anti-pTau (Ser202, Thr205) AT8 (1:1000; Invitrogen/Thermo Fisher Scientific; Mn1020), mouse anti-pTau (Ser214, Thr212) AT100 (1:5000; Invitrogen/Fisher Scientific, Waltham, MN1060) and rabbit anti-CD68 [RM1031] (1:2500; Abcam, ab303565). DAB images were captured at 10× (Echo Revolve R4 FL; Echo Laboratories, San Diego, CA) and at 40× (EasyScan One, Motic, Xiamen, China).

### Tau pathology type

Tau pathology type analysis was conducted by 3 independent and blinded investigators. Tau pathologies were defined based on previous publications [42–45] and based on the pattern of AT8 staining. Briefly as described by Shi et al. [43], type 1 displays mossy fiber as well as sparse and diffuse cell body staining in the dentate gyrus (DG), type 2 displays compact dense tangle-like cell body staining in the DG and CA3 with some sparse CA1 cell body staining, type 3 shows staining in the neuropil of the stratum radiatum of the CA1 along with staining of dendrites from pyramidal neurons and sparse cell body staining, and type 4 displays dense fragmented, dotted, and grainy staining all over the hippocampus. The percentage of pathology for the cohort for each type was determined by the following calculation: (number of mice with pathology type)/(total number of mice in cohort) × 100%.

### Image quantification

All analyses were conducted with either Qupath [46, 47] or ImageJ (FIJI) [48].

NeuN quantifications were done using QuPath cell detection. Identical ROIs were applied to each image for all 3 regions of the hippocampus. The cell detection tool was used to automatically detect NeuN^+^ cells based on identical thresholding settings applied to all images.

Quantification of Iba1^+^ and GFAP^+^ cells was conducted as previously described [40, 41]. Average projections of 20× tiled z-stack confocal images were generated in FIJI for analysis. Two ROIs (~3900 μm^2^) were drawn within the CA1, CA3, and DG and cells were manually counted using FIJI cell counter. Cells were classified as Iba1^+^ or GFAP^+^ when DAPI nuclei overlapped with the stained area. Counts were averaged and normalized per mm^2^ and reported as animal averages (2 ROIs/slice; 3 slices/animal).

DAB-positive percent area was quantified using either the Colordevonvolution2 plugin in FIJI [47] or in QuPath. Briefly, a threshold of positive DAB staining was determined and applied to all images to then calculate the percentage of positive area.

Quantification of PSD-95^+^ puncta within Iba1^+^ microglia was performed using Imaris software (Bitplane). A confocal microscope (Stellaris, Lecia) was used to acquire images in the CA1 stratum radiatum region of the hippocampus with a 40× objective and 3× zoom factor with 0.34 size z-steps for a total of 15 steps. Lecia Stellaris software lighting processing was applied before analysis. Images were then 3D rendered in Imaris and cropped to isolate individual Iba1^+^ cells (2 per slice). Iba1^+^ surfaces were generated and used to mask PSD-95^+^ signal. The masked PSD-95^+^ signal was then used to create spots, to ensure it was located within Iba1^+^ signal. The number of spots within the Iba1^+^ cell soma, defined as the region where the Iba1^+^ signal colocalized with DAPI, as well as within the total Iba1^+^ cell area, was quantified and reported as spot number per cell.

### Microglia morphology

Microglia morphology was quantified as described [49]*.* Iba1-stained slices were imaged using a Lecia Stellaris confocal microscope at 40× (2.5× zoom), and z-stacks were acquired at a 0.3-μm step size. Nine cells per animal (3 slices × 3 cells/slice) were analyzed. Maximum projection was processed in FIJI: brightness/contrast adjusted, converted to binary, and despeckled to remove noise and then skeletonized. The AnalyzeSkeleton plugin [50] was used for quantification.

### In situ hybridization

Tissues were collected as previously described in the immunohistochemistry procedure. Slices were mounted and stored at −80 °C overnight before in situ hybridization. RNAscope assay (ACDbio, Newark, CA) was performed according to the manufacturer’s protocol using a custom probe for *Csnk2a2*. Immediately following RNAScope protocol, the slides were blocked with 10% NGS in the Co-detection antibody diluent (CDD) for 1 h. The slides were incubated overnight with rabbit anti-Iba1 (Fujifilm Wako, Richmond, VA, 019-19741) diluted at 1:200 in 5% NGS with CDD. The slides were incubated the next day with Alexa-fluorophore-conjugated antibodies (Invitrogen/Thermo Fisher Scientific) (1:40 in CDD with 5% NGS) for 2 h at room temperature. The slides were mounted in ProLong Gold Antifade with DAPI (Invitrogen/Thermo Fisher Scientific) and subsequently imaged.

### Synapse analysis

Synapse analysis was conducted as previously described [14, 41, 51–53]. Immunohistochemical staining was conducted as described above with VGlut1 and PSD-95, with blocking increased to 20% NGS + TBST for 2 h and primary and secondary antibodies diluted in 10% NGS + TBST. Fluorescent images from the molecular layer of the CA1 were taken on a confocal microscope (Stellaris, Lecia) at 63× with z-step dimension of 0.34 μm for 15 steps. Lecia stellaris software lightning processing was applied to images post-acquisition and edited images were used for analysis. Maximum projections of three sections per slice were generated. Puncta analyses were conducted in a blinded manner using the PunctaAnalyzer Plugin (Durham, NC) in FIJI as previously described [14, 41, 51–53]. Data are presented as average from three slices per animal.

### Electrophysiology

Mice were anesthetized with isoflurane (2%) and decapitated for slice preparation. Brains were rapidly removed into the solution containing sucrose 189 mM, glucose 10 mM, NaHCO_3_ 26 mM, KCl 3 mM, MgSO_4_ 5 mM, CaCl_2_ 0.1 mM, and NaH_2_PO_4_ 1.25 mM. Transverse hippocampal slices (350 μm) were cut on a vibratome (LEICA VT1000S) and incubated at room temperature in oxygenated artificial cerebrospinal fluid (aCSF; NaCl 124 mM, KCl 5 mM, NaH_2_PO_4_ 1.25 mM, MgSO_4_ 2 mM, NaHCO_3_ 26 mM, CaCl_2_ 2 mM, glucose 10 mM; gassed with 95% O_2_, 5% CO_2_; pH = 7.4) for 45 min to 1 h. Experiments were performed at 30–34 °C with continuous perfusion.

Field excitatory postsynaptic potentials (fEPSPs) were recorded in the CA1 region of the hippocampus. fEPSPs were evoked with a stimulating electrode placed on the Schaffer collateral (0.33 Hz) using brief current pulses (200 μs, 0.1–0.2 mA). Extracellular recording electrodes were filled with aCSF. Stimulation was adjusted to obtain a fEPSP peak amplitude of approximately 1 mV during control conditions. After a stable fEPSP baseline period of 10 min, long-term potentiation (LTP) was induced by a theta-burst stimulation (TBS) consisting of a series of 10 bursts of 5 stimuli (100 Hz within the burst, 200 ms interburst interval), which was repeated 4 times (5 s apart). Data were filtered at 3 kHz and acquired at 10 kHz using pCLAMP 10.2 software (Molecular Devices, San Jose, CA; RRID: SCR_011323).

A stimulus–response curve (0.05–0.4 mV, mean of five fEPSPs at each stimulation strength) was compiled for the different mice used. For paired-pulse ratio (PPR) experiments, two fEPSPs were evoked 40 ms apart for 0.5 min at baseline frequency (6 times) at the beginning of the baseline recording. PPR was expressed as the amplitude of the second fEPSP divided by the amplitude of the first fEPSP.

### Behavior

Behavioral testing was performed with the support from the University of Minnesota Mouse Behavior Core. Mice were handled daily for ≥1 week and habituated to testing room ≥30 min before tasks. All tasks were recorded using the ANYmaze software (Stoelting Co., Wood Dale, IL) and overhead cameras.

Barnes maze was conducted on a 20-hole Barnes Maze (San Diego Instruments, San Diego, CA) under 450–500 lx illumination with black and white visual cues around the room, as described [54–56]. Mice were placed in the center under a cover with lights off. Lights were turned on and the cover removed within 30 s. Training consisted of four 3-min trials per mouse for 5 days, with 20–30 min of intertrial intervals. The maze was cleaned with 70% ethanol between mice and rotated between trials. Primary latency, or the time to first explore the escape hole, was recorded. Trials ended when mice entered the escape box or after 3 min, at which point the mouse was gently guided to the escape box. During the probe trial, the escape hole was covered and time spent in each maze zone was recorded. Spatial strategy was determined by a blinded investigator.

Open field was conducted in 50.8 × 50.8 × 25.4 cm^3^ boxes under 150-200 lx illumination. Mice were placed in boxes and behavior was recorded for 1 h. Boxes were thoroughly cleaned between mice with 70% ethanol.

### RNA extraction and qPCR

RNA was extracted from cells and mouse striatal tissues using the RNeasy extraction kit (Qiagen) and reverse-transcribed with SuperScript First Strand Synthesis System (Invitrogen/Thermo Fisher Scientific). SYBR green-based PCR was performed with qPCRBIO SyGreen Blue Mix Lo-ROZ (PCR Biosystems, London, UK; 17-505B) using the LightCycler 480 System (Roche, Basel, Switzerland). Samples were run in triplicate and normalized to GAPDH.

For qPCR the following primers were used: CK2α′ forward CGACTGATTGATTGGGGTCT, reverse AGAATGGCTCCTTTCGGAAT; CK2α forward TCCCCATGCTGTGACAATAA, reverse AAGACCCTGTGTCACGAACC; CK2β forward AGTCCTCCAGACACCACCAC, reverse GACTGGGCTCTTGAAGTTGC; huTau forward GCTGGCCTGAAAGCTGAAGA, reverse CGTTTTACCATCAGCCCCCT.

### RNA-Seq analysis

RNA-sequencing was conducted by Novogene (Sacramento, CA). Gene expression analysis was carried out with the CHURP pipeline (10.1145/3332186.3333156) using 4–7 mice/genotype with female (F)/male (M) ratios as follows: 4 WT (2F/2M), 5 CK2α′^(+/−)^ (2F/3M), 7 PS19 (3F/4M) (3 high pathology and 4 low pathology), and 4 PS19;CK2α′^(+/−)^ (3F/1M). Differential gene expression was determined with DESeq2 (v1.46.0) using default setting [57]. Genes with a FDR ≤ 0.05 were considered significant. Outliers’ identification was performed using Cook’s distance (DESeq2). Driver factors of gene expression variance (genotype and/or sex) were evaluated using R (v4.4.2) package variancePartition (1.36.3). Pathway and clustering analysis were completed with gProfiler2 [58] (v0.2.3) and clusterProfiler (v4.14.6). Data visualization was done using various R graphic packages, including ggplot2, ggraph, and DESeq2 visualization functions. To assess the cellular distribution of gene markers, we conducted cell-type annotation of the 215 gene list using the DropViz Hippocampus reference [59]. Each gene was categorized by its maximum normalized expression across the reference Metacells. The RNA-seq data set generated in this manuscript has been deposited at GEO (accession number GSE298505). The reviewer token to access the GEO deposited data is mlwxmuactlibryl.

### Statistical analyses and data representation

For electrophysiology experiments, data were analyzed using Clampfit 10.2 software (pCLAMP, Molecular Devices, RRID: SCR_011323), and presented as mean ± SEM. To estimate changes in synaptic efficacy, short-term potentiation (STP) was quantified by comparing the mean fEPSP amplitude during 1 min after application of TBS. LTP was quantified as for STP but 40 min after the protocol. Graphs were made using SigmaPlot 14.0. Before applying any statistical comparison, the data were subjected to Shapiro-Wilk normality and equal variance tests. For any comparisons between two groups, two-paired Student’s* t*-test was used. For multiple comparisons to the same control, one-way ANOVA and Holm-Sidak test were used. *P* < 0.05 was considered statistically significant.

For all other experiments, GraphPad Prism (GraphPad, San Diego, CA) was used to create graphs and conduct statical testing. For all other experiments, data are presented as mean ± SEM. Statistical analyses were performed using paired Student’s *t*-test, unpaired student’s* t*-test, one-way or two-way ANOVA with post-hoc analysis, as indicated in each figure legend. *P* < 0.05 was considered significant.

## Results

### Levels of protein kinase CK2α′ are increased in the brains of dementia patients and mouse models of tauopathy

**Fig. 1:**
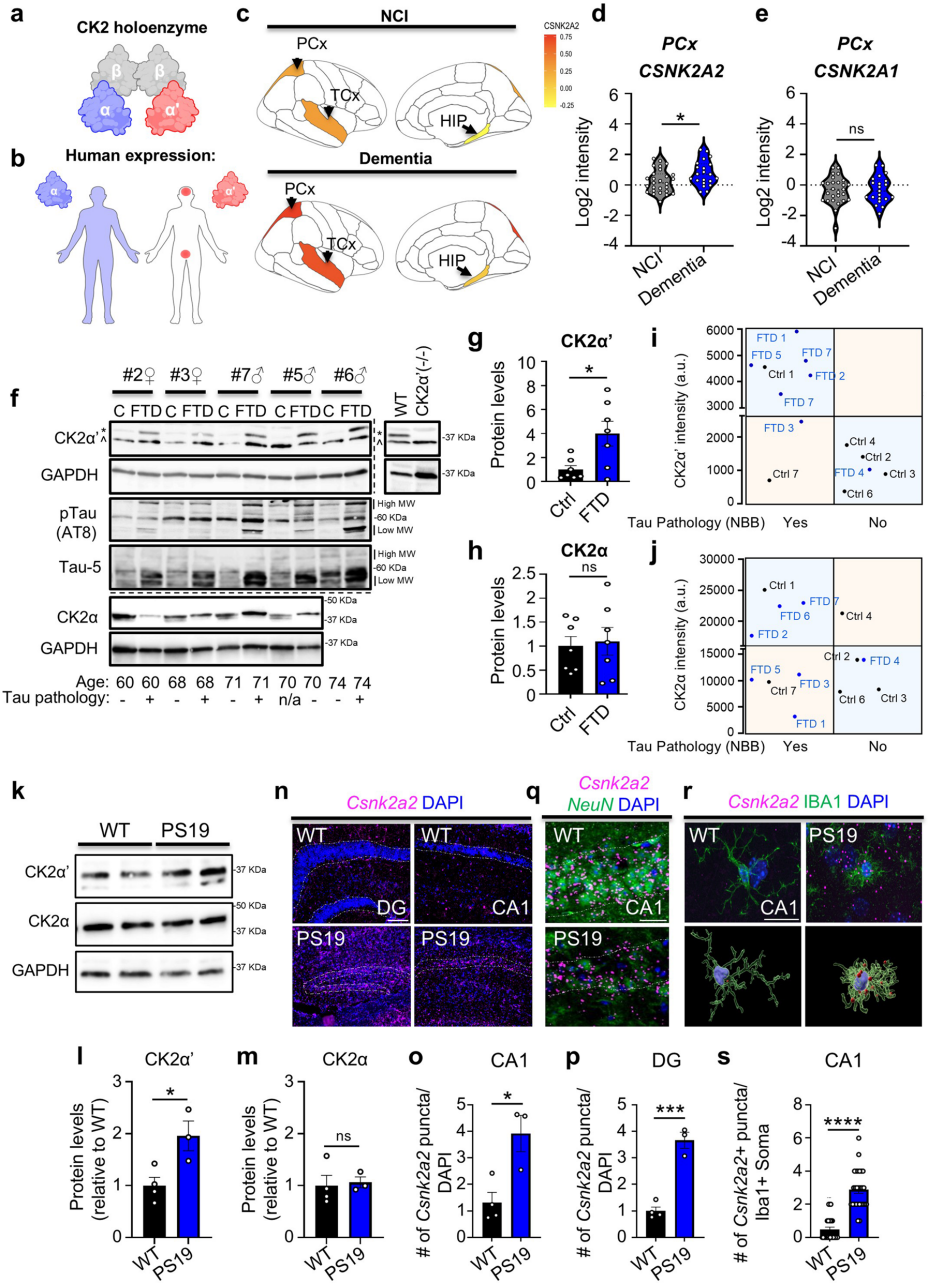
CK2α′ is increased in tissues from patients with AD/ADRD and in PS19 mice. **a** and **b** Representative diagrams of CK2 holoenzyme, including regulatory and catalytic subunits and differential distribution of catalytic subunits CK2α and CK2α′ in the human body. Images were created with BioRender.com. **c** Averaged expression of CSNK2A2 in non-cognitively impaired (NCI) and dementia samples from the Allen Brain Institute: Aging, Dementia and TBI study in the parietal cortex (PCx), temporal cortex (TCx), and hippocampus (HIP). Patients with TBI were excluded from the analyses. **d** and **e** Quantification of the expression of *CSNK2A2* and *CSNK2A1* in the PCx from NCI and dementia from the Allen Brain Institute: Aging, Dementia and TBI study (*n* = 20–27 patients/group). Significance determined by unpaired *t*-test, *P < 0.05. **f** Immunoblots for CK2α′, CK2α, total tau (Tau-5), and pTau (AT8) in the soluble protein lysates from the frontal cortex of age- and sex-matched healthy controls (C) and patients with FTD. Arrow indicates CK2α′-specific band, asterisk indicates unspecific band. Right panel, immunoblots for CK2α′ in WT and CK2α′^(−/−)^ mice showing antibody-band specificity. **g** and **h** CK2α′ and CK2α protein levels normalized to GAPDH and shown relative to age/sex-matched controls (*n* = 7 patients/group). Statistical analyses were conducted using unpaired *t-*test with welch’s correction. *P = 0.0377. Data are shown as mean ± SEM. **i** and **j** Relationship between the levels of CK2α′ (**i**) and CK2α (**j**) and the presence of tau pathology reported by the NIH biobank (Additional file 2). Significance determined by fisher exact test. *P* = 0.020979 (**i**) and *P* = 0.564880 (**j**). **k–m** Immunoblots for CK2α′ and CK2α in the hippocampus of symptomatic WT and PS19 mice, and quantification (*n* = 3 mice/genotype). **n** *Csnk2a2* (CK2α′) in situ hybridization in hippocampal cell layers of symptomatic WT and PS19 mice. Scale bar, 50 μm. **o** and **p** Quantification of Csnk2a2 puncta in the CA1 (*P* = 0.0163) (**o**), and DG (*P* = 0.0003) (**p**) quantified within the pyramidal cell layer identified by DAPI (*n* = 3–4 mice/genotype). **q** *Csnk2a2* in situ hybridization and NeuN immunofluorescence in CA1 of symptomatic WT and PS19. Scale bar, 20 μm. **r** *Csnk2a2* RNA in situ hybridization and Iba1 immunofluorescence in symptomatic WT and PS19 hippocampus. Top: representative images of a single Iba1^+^ cell in the Stratum Radiatum of the CA1. Scale bar, 20 μm. Bottom: 3D rendering of Iba1^+^ cells containing *Csnk2a2* puncta. **s** Quantification of the number of Csnk2a2^+^ puncta/Iba1^+^ soma (*P* < 0.0001, *n* = 3–4 mice/group, 8–9 cells/mouse, every data point represents a cell). All data shown are mean ± SEM. Unpaired *t*-test, *P < 0.05, **P < 0.01, ***P < 0.001, ****P < 0.0001. *P*-values can be found in Additional file 5

There are two different genes (*Csnk2a1* and *Csnk2a2*) that code for two different CK2 catalytic subunits CK2α and CK2α′, respectively. These two catalytic subunits share a high percent of homology and they can arrange in various combinations (α–α, α–α′ or α′–α′) depending on their expression and tissue availability, although the canonical arrangement in the brain is expected to be α–α′ (Fig. 1a, b) [13, 26–32]. By analyzing existing RNA-seq data from the Allen Brain Institute: Aging, Dementia, and Traumatic Brain Injury (TBI) Study [60], we found a specific increase in the expression of *CSNK2a2* (CK2α′), but not in *CSNK2a1* (CK2α), in the parietal cortex of patients with dementia compared to non-dementia controls (Fig. 1c–e). We also found a trend towards increased CK2α′ in the temporal cortex (TCx) and hippocampus (HIP) although the difference did not reach statistical significance (Fig. 1c) [60]. Importantly, we also observed a significant increase in CK2α′ protein level, but not CK2α, in the frontal cortex of FTD patients compared to age- and sex-match controls (Fig. 1f–h, Additional file 2). An apparent gel up-shift was also observed in CK2α immunoblot for FTD #5 and #7 samples compared to controls, which imply alterations in posttranslational modifications for this catalytic subunit. However, this gel upshift was similar to that seen in its corresponding GAPDH blot and was not consistent across all analyzed FTD samples (Additional file 3), being ultimately attributed to technical artifacts in the SDS–PAGE. Immunoblotting for CK2α′ in CK2α′ knock-out mice and immunoprecipitation experiments confirmed antibody specificity (Figs. 1f, S1a).

To assess the relationship between CK2α′ and tauopathy, we first assessed total tau levels (Tau-5 antibody) and pTau (AT8 antibody) in FTD and control samples (Fig. 1f). We observed an enhanced multiband pattern of Tau-5 in FTD samples, which likely reflects disease-associated post-translational modifications, isoform heterogeneity, and/or proteolytic processing in AD/ADRD patients [61–63]. In contrast, control samples displayed a more discrete tau band profile. Correlation analyses of total tau levels revealed a trend towards a significant positive correlation with both CK2α′ (*r*^2^ = 0.2826, *P* = 0.0753) and CK2α (*r*^2^ = 0.3110, *P* = 0.0595) (Fig. S1b, c). AT8 signal also showed a multiband profile in most FTD samples. Importantly, high molecular weight (MW) AT8 signal showed a trend towards positive correlation with CK2α′ (*r*^2^ = 0.2970, *P* = 0.0669) but not with CK2α (*r*^2^ =  − 0.0508, *P* = 0.4809), and low MW AT8 signal showed a significant positive correlation with CK2α′ (*r*^2^ = 0.4396, *P* = 0.0188) but not with CK2α (*r*^2^ = 0.2006, *P* = 0.1442) (Fig. S1d–i). The correlation between CK2α′ and pTau was further confirmed using a stratification of samples based on the presence of AT8 aggregates assigned by the NeuroBioBank (Fig. 1i, j, Additional file 2). This revealed that higher levels of CK2α′ were associated with AT8-positive cases, whereas AT8-negative cases clustered at lower CK2α′ levels (Fig. 1i; *P* = 0.02098). In contrast, CK2α levels did not show significantly different clustering based on AT8 positivity (Fig. 1j; *P* = 0.5649). Together, these findings indicate that while both CK2 subunits correlate with overall tau abundance, CK2α′ is uniquely elevated in disease (Fig. 1d, f, g) and is specifically enriched in AT8-positive cases (Figs. 1f, i, S1d, e). These data support a disease-associated role for CK2α′ in tauopathy.

To examine whether CK2α′ was also elevated in mouse models of tauopathy, we utilized the PS19 mouse model, which expresses human tau with a P301S mutation associated with familial forms of FTD and other tauopathies [39]. Pathology and neuronal loss in PS19 mice are seen largely in the hippocampus, although they can spread to other brain regions including the neocortex, entorhinal cortex and amygdala [39, 64]. CK2α′, but not CK2α, was found elevated in the hippocampus of symptomatic PS19 mice (10–12 months) [39, 65, 66] by both immunoblotting and RNA in situ hybridization (Fig. 1k–s). *Csnk2a2* RNA probe specificity was confirmed in CK2α′ KO mice, where no signal was observed (Fig. S1j). In PS19 mice, CK2α′ was increased in all regions of the hippocampus, especially in the pryamidal cell layer of CA1 and DG regions, where the signal colocalized with NeuN staining (Fig. 1n–q). Enhanced expression of CK2α′ was also observed outside the neuronal layer in microglia (Iba1+) (Fig. 1r, s). Single-cell RNA-Seq studies available in The Alzheimer’s Cell Atlas (TACA) [67–71] confirmed increased *CSNK2a2* expression in patients with AD in both microglia and neurons in the occipital cortex and entorhinal cortex, respectively (Fig. S2). However, studies in prefrontal cortex and superior frontal gyrus within the frontal lobe revealed enhanced expression of *CSNK2a2* in cells other than neurons and microglia, suggesting a cell-specific upregulation of *CSNK2a2* that seems to be brain region-dependent. Overall, these results demonstrate a specific upregulation of CK2α′ in affected brain regions of patients with dementia and in hippocampal neurons and microglia of mouse models of tauopathy.

### Genetic depletion of CK2α′ impacts tau phosphorylation and pathology in vitro and in vivo

**Fig. 2:**
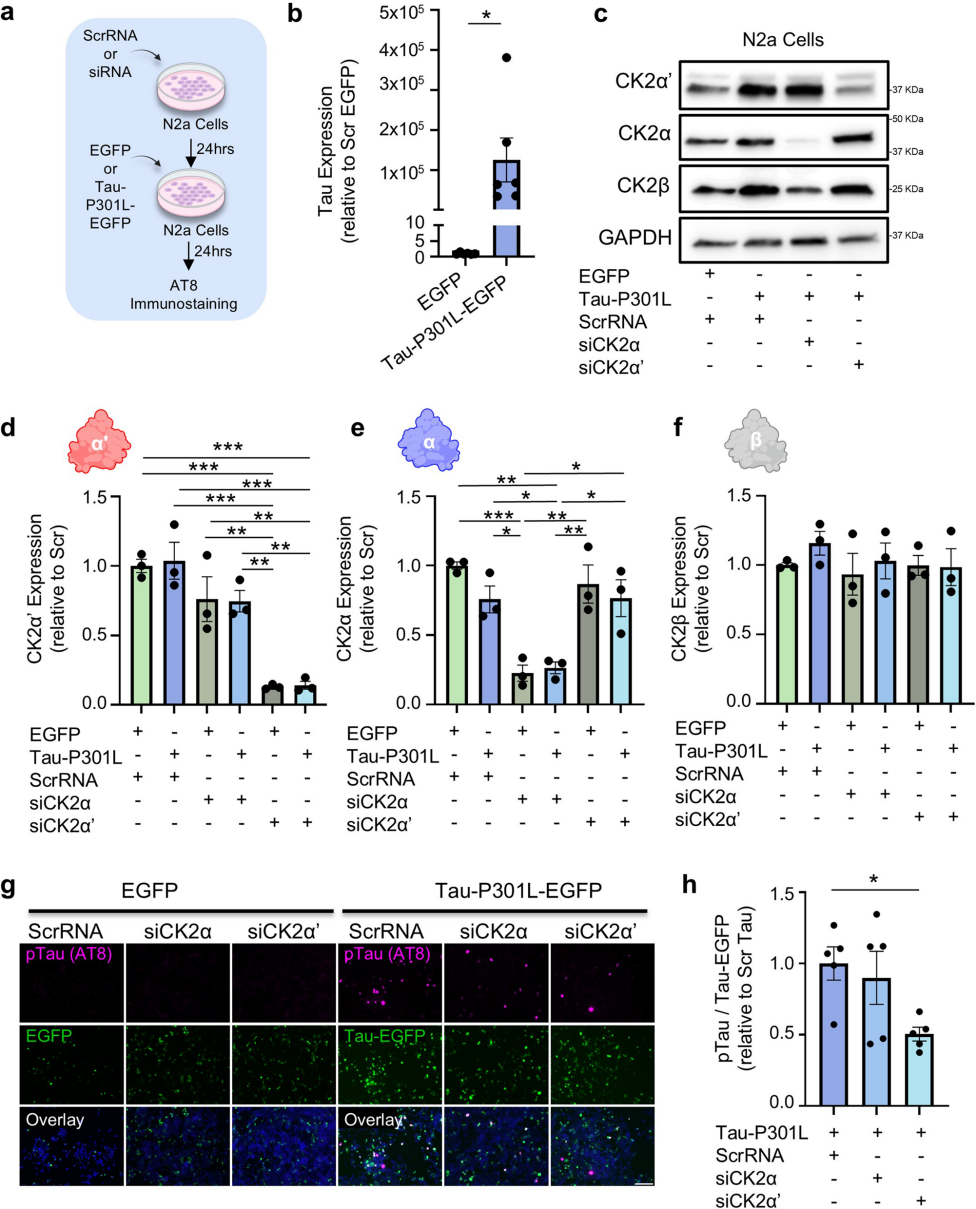
Silencing CK2α′ reduces pTau levels in N2a cells expressing Tau-P301L.** a** Schematic of experimental design for N2a cell transfection and immunostaining. **b** RT-qPCR analysis of mRNA expression levels of hTau in N2a cells transfected with EGFP or Tau-P301L-EGFP. Expression levels were normalized to GAPDH and presented relative to EGFP condition (*P* = 0.0464, *n* = 6/condition). **c** Immunoblots of CK2α′, CK2α and CK2β in N2A cells transfected with EGFP or Tau-P301L-EGFP and treated with siCK2α′ or siCK2α. **d**–**f** RT-qPCR analysis of mRNA expression levels of CK2α′ (**d**)**,** CK2α (**e**), and CK2β (**f**) in transfected N2a cells. Expression levels were normalized to GAPDH and presented relative to ScrRNA-EGFP condition (*n* = 3 independent replicates). **g** Representative images of AT8 immunostaining and EGFP fluorescence in N2a cells transfected with either EGFP or Tau-P301L-EGFP and treated with ScrRNA, siCK2α, or siCK2α′. Scale bar, 100 μm.** h** Quantification of AT8^+^ cells normalized to the number of EGFP^+^ cells, presented relative to the ScrRNA-Tau-P301L condition (*n* = 4/condition; Scr-Tau-P301L vs. siCK2α′-Tau-P301L *P* = 0.0472). All data shown as mean ± SEM. Statistical analyses were conducted using one-way ANOVA with Tukey’s post-hoc test, *P < 0.05, **P < 0.01, ***P < 0.001. *P*-values can be found in Additional file 5

Previous studies have shown that CK2 holoenzyme regulates tau phosphorylation in various cell and mouse models of AD [18, 24], but whether the CK2α′ subunit specifically contributes to this phenomenon is unknown. We first explored the impact of silencing CK2α or CK2α′ on tau phosphorylation in Neuro2a (N2a) cells expressing a pathological tau variant (Tau-P301L). N2a cells were treated with ScrRNA, siCK2α or siCK2α′ and transfected with either Tau-P301L-EGFP [72] or EGFP empty vector (control) (Fig. 2a). First, we confirmed the increased expression of hTau in N2A transfected cells (Fig. 2b) and the efficacy of the siRNAs decreasing the expression of CK2α or CK2α′ without altering the other subunits (Fig. 2c–f). Importantly, siCK2α′ (*P* = 0.0472), but not siCK2α (*P* = 0.8482), reduced AT8 levels in Tau-P301L transfected cells (Fig. 2g, h), demonstrating an impact of the CK2α′ subunit on pTau accumulation in vitro.

**Fig. 3:**
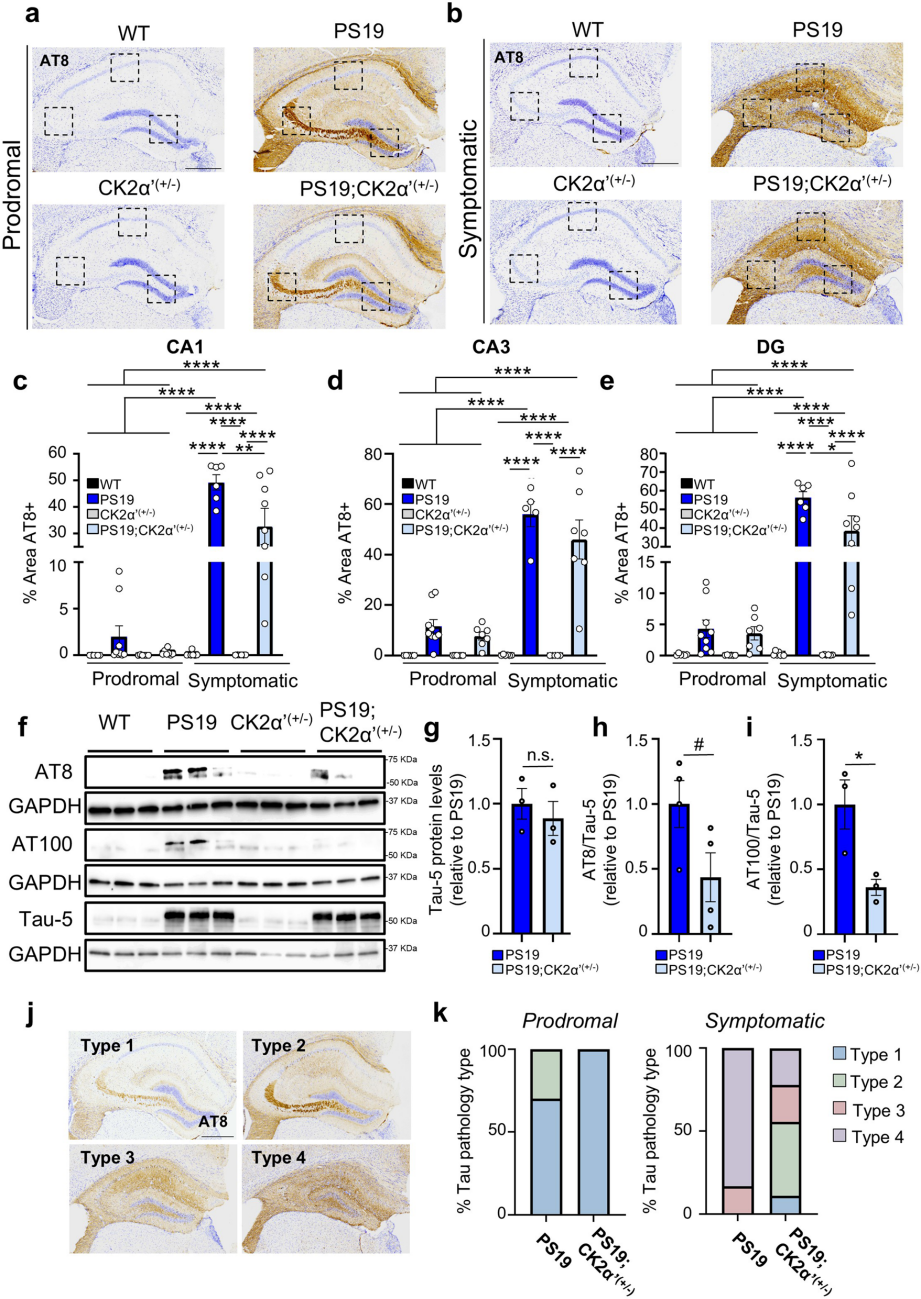
CK2α′ haploinsufficiency reduced tau pathology in PS19 mice. **a** and **b** Representative hippocampal sections immunostained for pTau (AT8; Ser202/Thr205). Scale bars, 500 μm.** c–e** Quantification of AT8^+^ immunoreactivity (% area) in the CA1 (**c**), CA3 (**d**), and DG (**e**) regions in prodromal and symptomatic cohorts (*n* = 6**–**9 mice/genotype). Data are presented as mean ± SEM. Two-way ANOVA with Tukey’s post hoc test, *P < 0.05, **P < 0.01, ***P < 0.001, **** *P* < 0.0001. **f** Immunoblots for AT8 (pS202/T205), AT100 (pT212/pS214), and Tau-5 (Total tau) in hippocampal samples obtained from 9-month mice. **g–i** Quantification of Tau-5 (**g**) *P* = 0.5578, AT8/Tau-5 (**h**) *P* = 0.0740, and AT100/Tau-5 (**i**) *P* = 0.0329 in** f** (*n* = 3–4 mice/genotype). All data are shown as mean ± SEM. Unpaired *t*-test, n.s. = non-significant (*P* > 0.1), ^#^*P* < 0.1, *P < 0.05. **j** Representative pTau pathology types. Scale bar, 500 μm. **k** Proportion of each pTau pathology type in prodromal and symptomatic cohorts (*n* = 7–10 mice/genotype). Fisher’s exact* t*-test, 7-month prodromal *P* = 0.11; 12-month symptomatic *P* = 0.0587. *P*-values can be found in Additional file 5

We then generated a PS19 mouse model haploinsufficient for CK2α′ (PS19;CK2α′^(+/−)^) (Fig. S3a–c) to assess the impact of CK2α′ deficiency on tau pathology in vivo at both a prodromal (~7 months) and a late symptomatic age (~10**–**12 months) [39] (Fig. 3a, b). In the prodromal stage, no significant differences were observed in the accumulation of AT8 between PS19 and PS19;CK2α′^(+/−)^ in the hippocampus (CA1, CA3, and DG) and overlaying cortex (Figs. 3c–e, S3d–f). As expected, at the late symptomatic group stage, we observed a significant increase in AT8^+^ area in both PS19 and PS19;CK2α′^(+/−)^ mice in the CA1, CA3, and DG (Fig. 3c–e) and cortex (Fig. S3d–f) relative to all other groups. However, the PS19;CK2α′^(+/−)^ mice showed a significant decrease of AT8 signal compared to PS19 in the CA1, DG, and cortex (CA1; *P* = 0.0075, DG; *P* = 0.0186 and Cortex; *P* < 0.0001) (Figs. 3c, e, S3d–f), suggesting an overall decrease in pTau upon depletion of CK2α′. Similar results were obtained using the AT100 phospho-tau antibody (Fig. S3g–i). Decreased pTau in PS19;CK2α′^(+/−)^ was also confirmed by immunoblotting from whole hippocampal samples at 9 months (Fig. 3f–i). Importantly, complete CK2α′ knockout in the PS19 (PS19;CK2α′^(−/−)^) did not confer a further decrease in pTau compared to PS19;CK2α′^(+/−)^ (Fig. S3j–n), perhaps due to the compensatory effects and/or off-target effects because of a complete removal of CK2α′. Since haploinsufficiency of CK2α′ was sufficient to significantly reduce pTau levels, with no additional benefits observed upon complete deletion, and as the CK2α′ levels were closer to physiological conditions (Fig. S3j, k), we focused our studies on the PS19:CK2α′ haploinsufficient model.

Tau pathology has been reported to follow a characteristic progressive pattern of deposition in the hippocampus that can be categorized into 4 pathology types [42–45] (Fig. 3j). These pathology types correlate with hippocampal volume [43, 45] and offer a more holistic pathological assessment. We observed differential distributions of patterns between PS19 and PS19;CK2α′^(+/−)^ mice, with a more dramatic alteration in late symptomatic stages when nearly all PS19 mice presented a type 4 pathology in contrast to the PS19;CK2α′^(+/−)^ mice with almost 50% presenting type 2 pathology (prodromal *P* = 0.11, and symptomatic *P* = 0.0587) (Fig. 3k). The results underscored a critical role for CK2α′ in the modulation of tau phosphorylation and associated pathology.

### RNA-Seq reveals differential gene expression associated with neuroimmune- and synaptic-related pathways in CK2α′ haploinsufficient PS19 mice

**Fig. 4:**
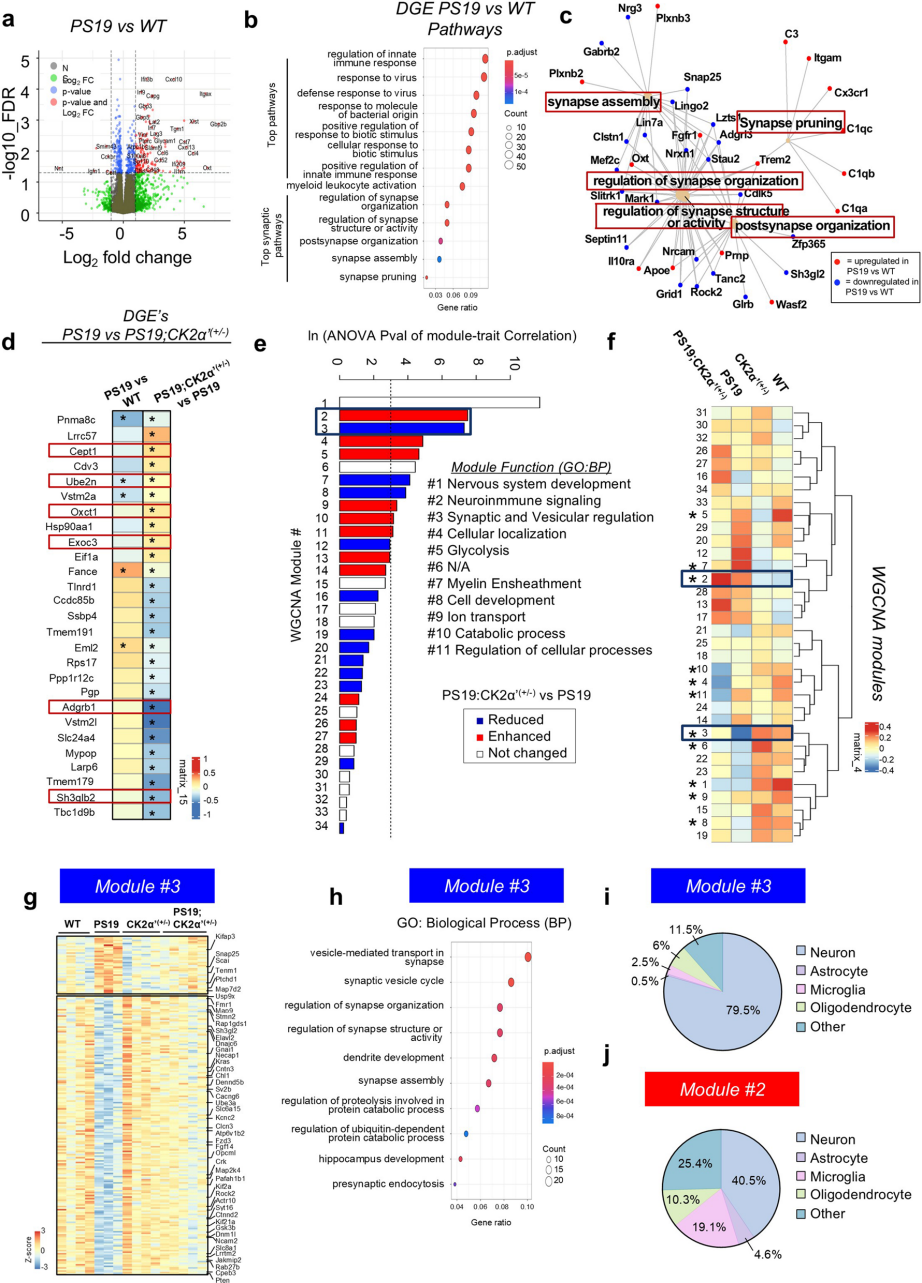
CK2α′ haploinsufficiency reduced transcriptional alterations in apoptotic/phagocytic and synaptic function-related genes in PS19 mice.** a** Volcano plot of DEGs in PS19 versus WT comparison (*n* = 3–4 mice/genotype). **b** Gene-ontology showing the top biological pathways for DEGs in PS19 vs WT. **c** Gene network for the top 5 synaptic pathways in PS19 versus WT. Red and blue dots indicate upregulated and down-regulated genes in PS19 mice, respectively. **d** Heat map displaying DEGs identified in PS19 versus PS19;CK2α′^(+/−)^ mice (*n* = 3–4 mice/genotype). Red boxes indicate genes with apoptotic/phagocytic and immune-related functions. *P < 0.05. **e** WGCNA-identified modules;  dotted line indicates significance as determined by the ANOVA test among WT, CK2α′^(+/−)^, PS19, and PS19;CK2α′^(+/−)^ mice (*n* = 3–5 mice/genotype). **f** Heatmap of modules detected from WGCNA indicating differences across genotypes based on z-scores. *P < 0.05 determined by the ANOVA test. **g** Heat map based on z-scores of the 215 genes of module #3. Selected 48 neuronal genes are shown. **h** Gene ontology for top biological pathways for module #3. **i** and **j** Distribution of genes into their associated CNS cell type for module #3 (**i**) and module #2 (**j**). Other cells include choroid plexus, endothelial, ependymal, and mural cells

To determine whether the reduction in tau pathology translates into wider changes in cellular signaling and gene regulation, we next analyzed RNA-seq profiles from the hippocampus of 7-month-old CK2α′ haploinsufficient mice compared to WT and PS19 mice. We focused on the hippocampus as a major area of pathology, and the region where we had previously demonstrated decreases in tau pathology. Additionally, we chose the prodromal phase to identify pathways involved in disease onset that may be missed if only later stages of the disease are studied where significant neuronal loss is present. Gene expression analyses in the PS19 versus WT mice identified 477 significant differentially expressed genes (DEGs) (Fig. 4a). Gene ontology (GO) pathway analysis revealed that many of the DEGs belonging to the top eight dysregulated pathways corresponded to immune and inflammatory functions (Fig. 4b), as previously reported [73, 74]. Other important GO pathways were associated with synaptic dysfunction where the top 5 corresponded to dysregulation in synapse assembly, regulation of synapse organization, regulation of synaptic structure or activity, post synapse organization and synaptic pruning (Fig. 4b). Synaptic pruning was upregulated in PS19 versus WT, whereas the other synaptic pathways related to organization and regulation of activity were largely downregulated (Fig. 4c).

Direct comparison of PS19 versus PS19;CK2α′^(+/−)^ mice yielded a small set of 26 significant DEGs (Fig. 4d). Several of these DEGs were related to Apoptotic/phagocytic and Immune functions (*Cept1, Ube2n, Oxct1, Exoc3, Adgrb1, Sh3glb2*). Most of the DEGs between PS19 and PS19;CK2α′^(+/−)^ were up-regulated in PS19 mice compared to WT but reduced in PS19;CK2α′^(+/−)^. For example, Adgrdb1 (Adhesion G protein–coupled receptor D1), which is strongly associated with the engulfment and pruning of synapses [75, 76], was significantly downregulated in PS19;CK2α′^(+/−)^ mice (Fig. 4d). We then assessed whether the manipulation of CK2α′ had a broader effect on modules of genes with similar biological functions despite not reaching the strict >Log2 criteria. WGNCA (weighted gene co-expression network analysis) revealed 34 gene modules, with 11 modules (#1–#11) showing statistical significance detected via ANOVA test among our groups (*P* < 0.05) (Fig. 4e, Additional file 3). Among the 11 significant gene modules, #3, #7 and #8 modules showed an expression pattern in the PS19;CK2α′^(+/−)^ mice that more closely resembled the expression pattern of WT mice, showing an overall rescue in expression compared to PS19 mice (Fig. 4e, f). These gene modules corresponded to genes involved in synaptic function and vesicular transport (#3), myelination and ensheathment (#7), and cell development (#8) based on GO:BP terms (Figs. 4e, f, S4). Two modules (#1 and #6) presented similar gene dysregulation between PS19 and PS19;CK2α′^(+/−)^ mice. The #1 module corresponded to genes involved in nervous system development, and the #6 did not provide any identified GO:BP term (Figs. 4e, f, S4). Finally, six modules presented a similar direction in the gene dysregulation in PS19 and PS19;CK2α′^(+/−)^ compared to WT mice, but were further dysregulated in PS19;CK2α′^(+/−)^ mice. Module #2 corresponded to stress response and neuroimmune signaling and was upregulated in PS19 and further enhanced in PS19;CK2α′^(+/−)^ mice (Figs. 4e, f, S4). On the contrary, modules #4, #5, #9, #10 and 11# that corresponded to cellular localization (#4), glycolytic processes (#5), metabolic and ion transport (#9), catabolic processes (#10) and regulation of cellular processes (#11), were down-regulated in PS19 and further downregulated in PS19;CK2α′^(+/−)^ mice (Figs. 4e, f, S4).

Module #3 was the most relevant module, based on its significance (ANOVA *P* = 0.000675) and rescue of its expression pattern by CK2α′ happloinsufficiency (Fig. 4e-g). Module #3 was composed of 215 genes (Additional file 3). The overall expression of genes associated with this module (~80%) presented a downregulation in the PS19 mice, which was ameliorated in the PS19;CK2α′^(+/−)^ group (Fig. 4g). This gene module was represented by pathways associated with synaptic function and assembly, with top pathways being vesicle-mediated transport in synapse, synaptic vesicle cycle, and regulation of synapse organization (Fig. 4h). Importantly, ~80% of genes in module #3 corresponded to neuron-associated genes (Fig. 4i). On the other hand, module #2 (2765 genes, *P* = 0.000553), the second most significant module in our data set, associated with stress response and neuroimmune signaling, contained ~20% microglia-associated genes and ~10% astrocyte-associated genes (Fig. 4j), with only ~40% genes associated with neuronal function. This module also contained genes involved in synaptic pruning like *Adgrb1* that belonged to DEGs identified in PS19;CK2α′^(+/−)^ mice compared to PS19. Overall, these results suggest amelioration in synapse pruning, improvement in synaptic function and organization, and an increase in stress response and neuroimmune signaling when deleting CK2α′ in PS19 mice.

### CK2α′ haploinsufficiency in PS19 mice ameliorates microglial reactivity, neuroinflammation and phagocytic activity

**Fig. 5:**
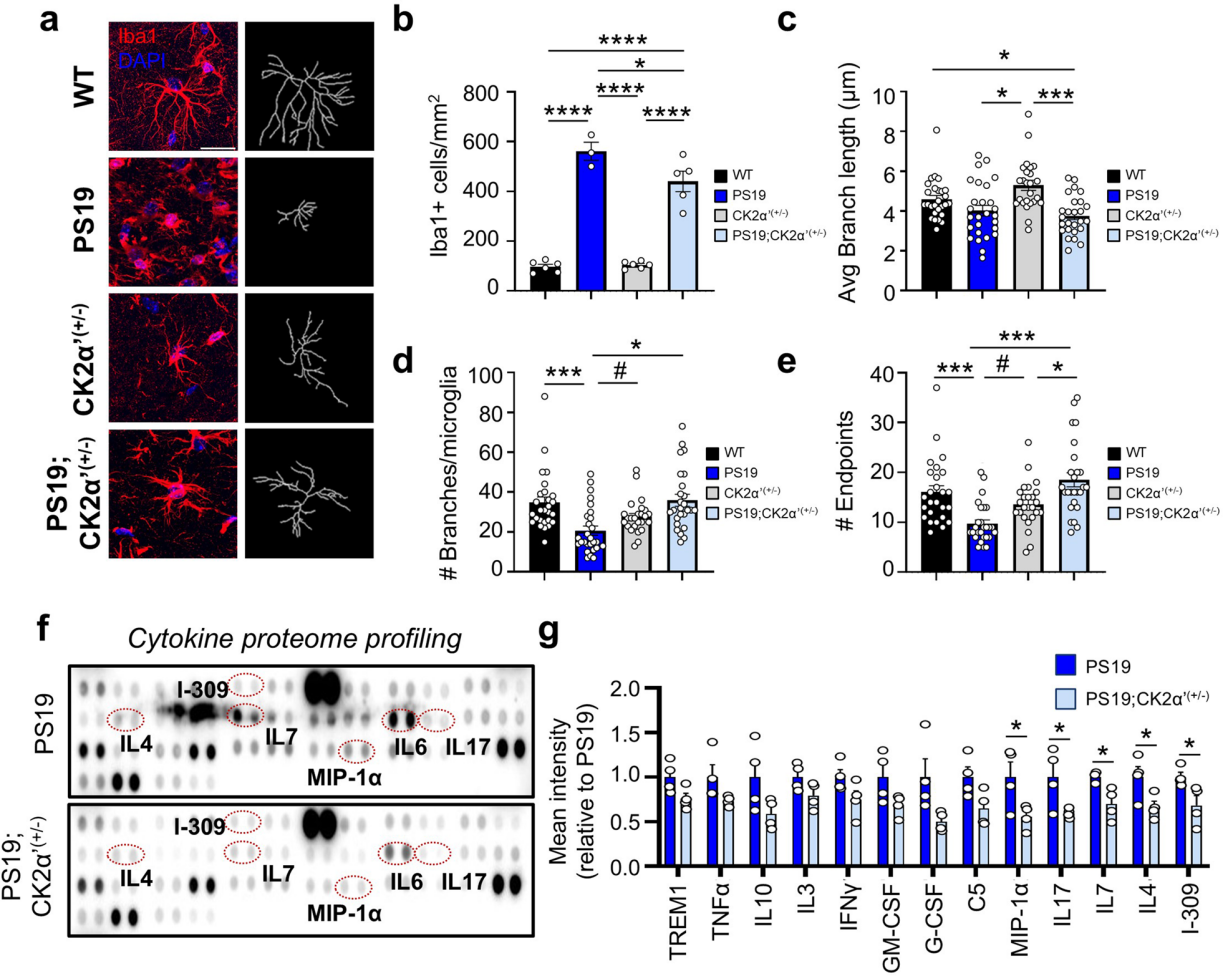
CK2α′ haploinsufficiency impacts the morphology of Iba1^+^ microglia and cytokine production in PS19 mice. **a** Iba1^+^ microglia immunostaining and microglia skeleton representation in the CA1 of symptomatic animals. Scale bar, 20 μm. **b** Quantification of the number of Iba1^+^ cells in the CA1 (*n* = 3–6 mice/genotype). **c–e** Quantification of microglial branch length (**c**), number of branches per microglia (**d**), and number of endpoints per microglia (**e**) (*n* = 27 cells (3 mice/genotype, 3 slices/mouse, 3 cells/slice). **f** Representative cytokine arrays using hippocampal protein extracts from PS19 and PS19;CK2α′^(+/−)^ at 9 months. Cytokines that showed significant differences are circled in red.** g** Quantifications of selected cytokines showing significance or trending significance. Quantifications are presented relative to PS19 (*n* = 4 mice/genotype). Data are shown as mean ± SEM. Statistical analyses were conducted using One-way ANOVA with Tukey’s post hoc test in **b** and Geisser-greenhouse correction and Tukey's post hoc test in **c–e** and unpaired *t*-test in **g,** ^#^*P* < 0.1, *P < 0.05, ****P < 0.0001. *P*-values can be found in Additional file 5

pTau is a potent driver of microglia reactivity [9, 39, 77–80] which in turn contributes to exacerbating tau-mediated pathology [73, 81–85]. We established that CK2α′ is elevated in PS19 microglia, and that deletion of CK2α′ decreased pTau accumulation and resulted in the downregulation of genes associated with apoptosis/phagocytosis and synaptic pruning and enhanced neuroimmune signaling. We therefore investigated whether these phenotypes were associated with changes in microglia reactivity. We first examined the number of Iba1^+^ microglia in the hippocampus of mice as an indicator of microgliosis (Figs. 5a, b, S5a–e). Iba1^+^ cells were significantly increased in PS19 mice compared to WT, especially in symptomatic mice. Importantly, the PS19;CK2α′^(+/−)^ mice displayed significantly reduced number of Iba1^+^ microglia in the CA1 and CA3 (Figs. 5a, b, S5a–e). Microgliosis was also reduced in the cortex of PS19;CK2α′^(+/−)^ mice compared to PS19 (Fig. S5f–h). We also analyzed the number of GFAP^+^ cells in the hippocampus of PS19 mice in both age groups (Fig. S6), as increased astrogliosis is also reported in PS19 mice [39]. However, no significant differences were found between PS19 and PS19;CK2α′^(+/−)^ mice (Fig. S6), suggesting that the CK2α′-mediated changes in glial cells are specific to microglia.

We then assessed ramification of microglia (Fig. 5a, c–e). It has been previously reported that microglia adopt an amoeboid morphology in PS19 mice, characterized by the loss of microglia processes, and that is associated with a more reactive and phagocytic state [9, 86]. Both PS19 and PS19;CK2α′^(+/−)^ mice showed a decrease in the average branch length of microglia in the CA1 (Fig. 5c). However, the PS19;CK2α′^(+/−)^ mice showed significant increases in the number of branches and the number of endpoints compared to the PS19 mice (Fig. 5d, e), indicating an improvement in the morphological state of microglia. Since the morphological state of microglia is strongly related to its reactive state and neuroinflammation, we first assessed the expression of cytokines and inflammatory molecules associated with microglial reactivity in hippocampal extracts from symptomatic PS19 and PS19;CK2α′^(+/−)^ mice, using a cytokine array panel (Fig. 5f, g). Importantly, we confirmed that several cytokines were significantly decreased (*P* < 0.05) in PS19;CK2α′^(+/−)^ mice compared to PS19, including MIP-1α, IL17, IL7, IL4, and I309 (Fig. 5f, g).

**Fig. 6:**
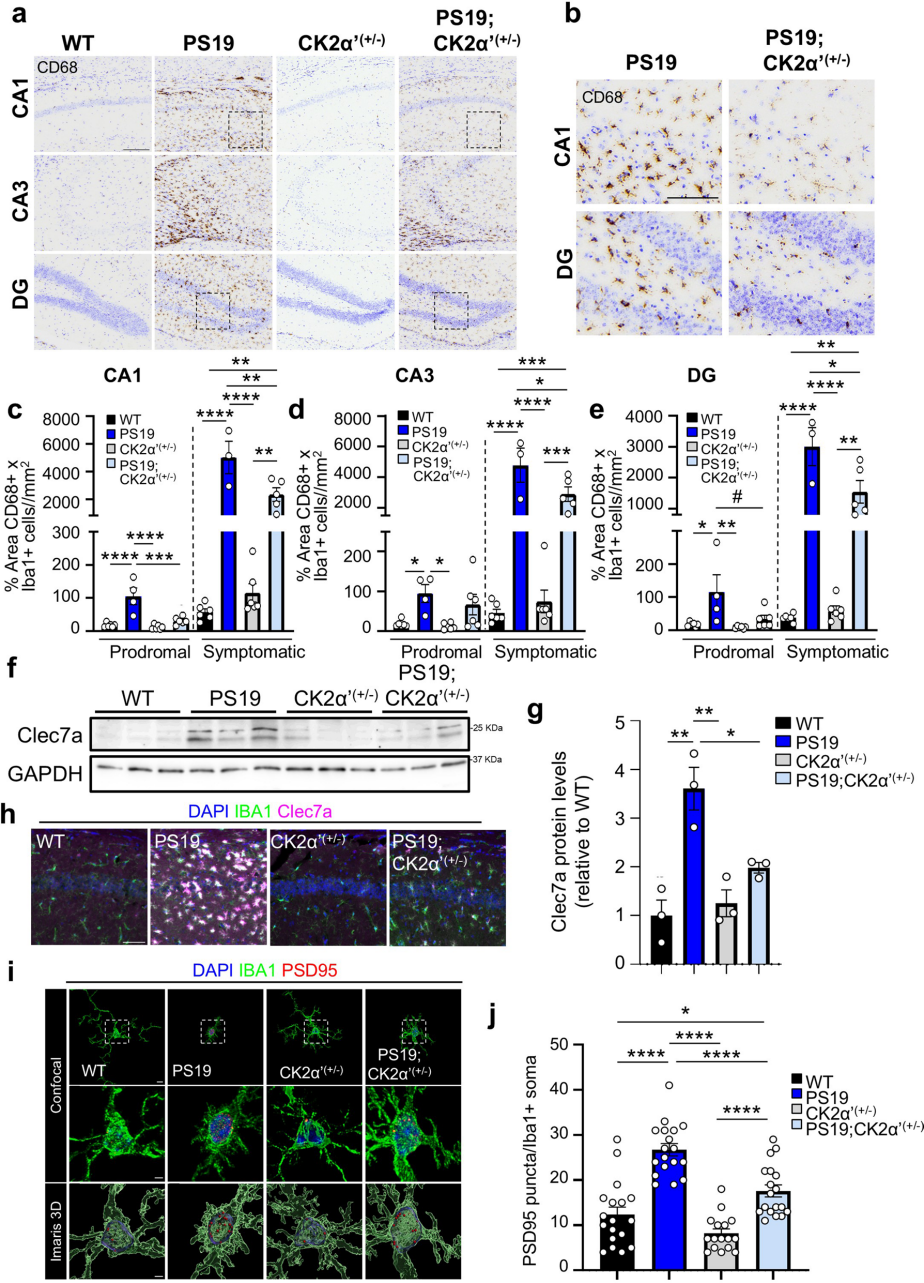
CK2α′ haploinsufficiency decreases microglial reactivity and synaptic engulfment. **a** CD68 immunostaining with cresyl violet counterstain in the hippocampus of symptomatic mice. Scale bar, 150 μm. **b** Zoomed images from **a** in CA1 and DG. Scale bar, 75 μm. **c–e** Quantifications of % area CD68^+^ normalized by Iba1^+^ cell count (shown as % Area CD68^+^ × Iba1^+^ cells/mm^2^) in prodromal and symptomatic animals in CA1, CA3 and DG (*n* = 3–6 mice/genotype). **f** and **g** Immunoblotting and quantification of protein levels of Clec7a in the hippocampus of symptomatic animals (*n* = 3 mice/genotype; WT vs. PS19 *P* = 0.0015, PS19 vs. CK2α′^(+/−)^ *P* = 0.0029, PS19 vs. PS19;CK2α′^(+/−)^ *P* = 0.0241). **h** Representative immunofluorescence of Clec7a in the hippocampus of symptomatic animals. **i** Iba1 and PSD95 immunostaining in the CA1 of symptomatic animals. Top row: whole microglia including processes; scale bar, 5 μm. Middle row: higher magnification view of the soma (white dotted box in top row); scale bar, 2 μm. Bottom row: 3D reconstruction of Iba1 and PSD-95 signal generated from Imaris Software; scale bar, 2 μm. **j** Quantification of the number of PSD-95 puncta/Iba1^+^ soma (*n* = 16–18 cells, 3 mice/genotype, 2–3 slices/mouse, 22 cells/slice). Data are shown as mean ± SEM. Statistical analyses were conducted using one-way ANOVA with Tukey’s post hoc test, *P < 0.05, **P < 0.01, ***P < 0.001,****P < 0.0001. *P*-values can be found in Additional file 5

We also investigated whether changes in the overall cytokine levels and microglial reactivity upon CK2α′ depletion were related to an altered phagocytic state. For this we assessed the level of CD68, a lysosomal/endosomal marker strongly associated with the phagocytic function of microglia, and normalized it to the number of Iba1^+^ cells (Figs. 6a–e, S5i). We observed increased levels of CD68 in all regions of the hippocampus in the PS19 mice at both prodromal and symptomatic stages (Fig. 6c–e). The PS19;CK2α′^(+/−)^ mice also presented increased CD68 levels compared to WT, but these levels were significantly decreased in the CA1 at the prodromal stage compared to PS19 mice (Fig. 6c). Importantly, the symptomatic PS19;CK2α′^(+/−)^ mice displayed significantly less CD68 in all hippocampal regions compared to PS19 mice (Fig. 6c–e). Expression of another phagocytic marker Clec7a was also significantly reduced in PS19;CK2α′^(+/−)^ compared to PS19 mice, supporting the impact of CK2α′ deletion on microglia phagocytosis (Fig. 6f–h).

Abnormalities of microglia-mediated synaptic pruning have been extensively reported in PS19 mice and contribute to synaptic dysfunction and neuronal loss [73, 81–85]. Considering the changes observed in CD68 and Clec7a, markers of microglia phagocytosis, and transcriptional changes related to apoptosis and synaptic pruning upon deletion of CK2α′, we assessed whether microglial synaptic engulfment was decreased in PS19;CK2α′^(+/−)^ mice. To quantify synaptic phagocytosis/engulfment, we co-stained for the postsynaptic marker PSD-95 and Iba1 [87, 88] and examined the CA1 stratum radiatum of symptomatic mice (Fig. 6i). PS19 mice exhibited a significant increase in PSD-95^+^ puncta within Iba1^+^ cells relative to control mice (Fig. 6i, j)**,** demonstrating increased phagocytosis. Importantly, the PS19;CK2α′^(+/−)^ mice showed a significant reduction in PSD-95 engulfment (Fig. 6i, j), indicating amelioration in the phagocytic activity of microglia upon deletion of CK2α′.

### CK2α′ happloinsufficiency improves hippocampal synaptic density and synaptic function in PS19 mice

**Fig. 7:**
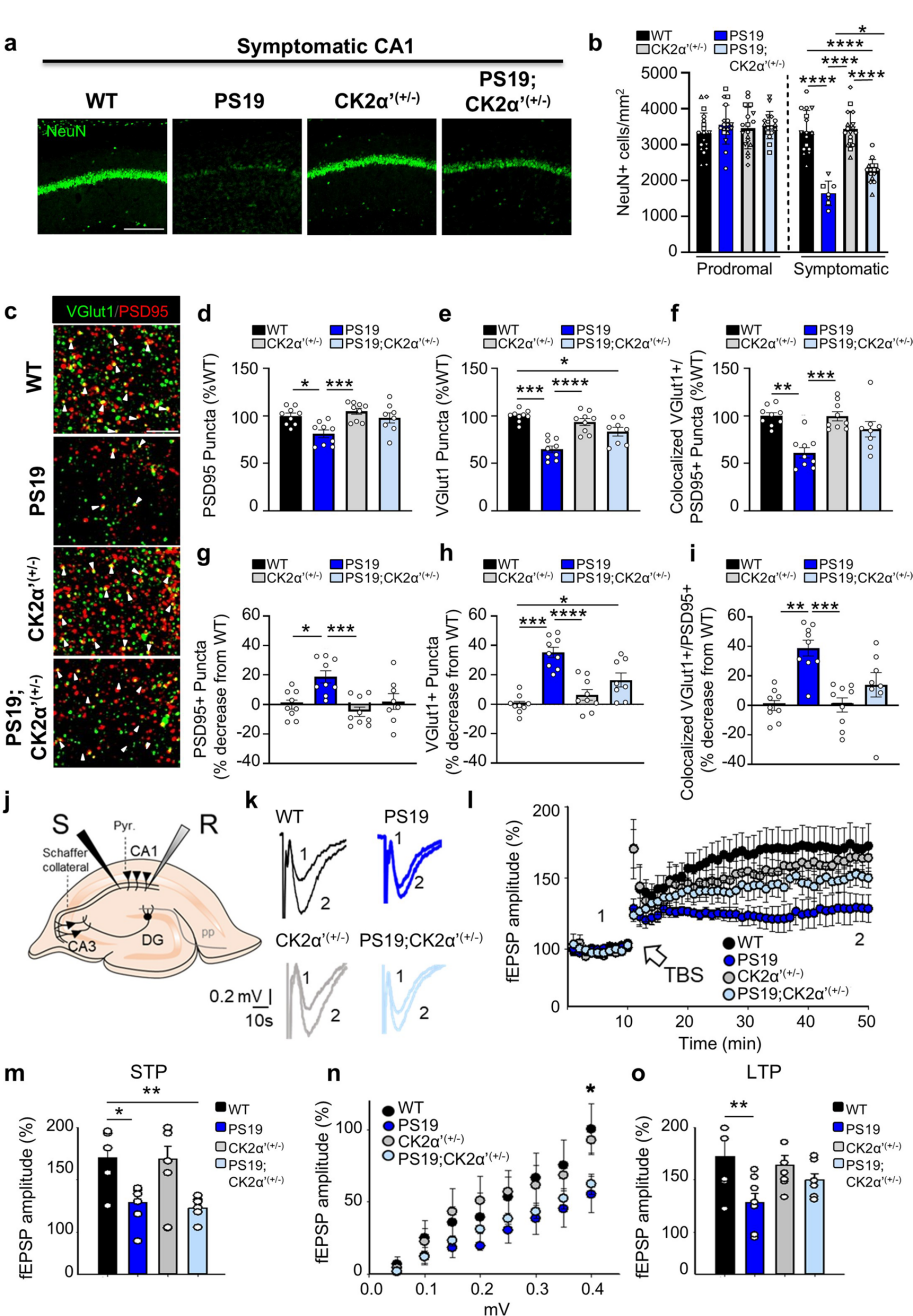
CK2α′ haploinsufficiency partially rescues hippocampal synaptic loss and improves LTP in PS19 mice. **a** Representative NeuN staining in the CA1 of symptomatic mice. Scale bar, 200 μm.** b** Quantification of NeuN^+^ cells (cells/mm^2^) in the CA1 of prodromal and symptomatic cohorts (*n* = 3–6 mice/genotype; 2–3 slices/mouse; each data point represents a slice, slices from the same animal share a shape). Comparisons shown relative to age group.** c** VGlut1 and PSD95 immunostaining in the CA1 of symptomatic mice. White arrow heads indicate colocalization. Scale bar, 2.5 μm. **d–f** Quantification of PSD95 puncta (WT vs. PS19 *P* = 0.0445, PS19 vs. CK2α′^(+/−)^ *P* = 0.0008) (**d**), VGlut1 puncta (WT vs. PS19 *P* = 0.0003, WT vs. PS19;CK2α′^(+/−)^ *P* = 0.0212, PS19 vs. CK2α′^(+/−)^ *P* < 0.0001) (**e**) and VGlut1/PSD95 colocalized puncta (WT vs. PS19 *P* = 0.0017, PS19 vs. CK2α′^(+/−)^ *P* = 0.0004) (**f**). *n* = 3 mice/genotype (3 slices/mouse, 3 images/slice, 1 data point = slice average). **g**–**i** Analyses from **d–f** represented as a % decrease from WT. **j** Experimental set-up: R, recording electrode; S stimulating electrode. **k** Representative traces before (1) and after (2) theta-burst stimulation (TBS). **l** Course temporal of TBS application inducing LTP in pyramidal neurons of CA1 (*n* = 6**–**7 mice/genotype). **m** Short-term potentiation (STP) results (WT vs. PS19 *P* = 0.014, WT vs. PS19;CK2α′^(+/−)^ *P* = 0.007).** n** Input–output curves. Averaged fEPSP amplitude (*n* = 3 mice/genotype). **o** LTP results (WT vs. PS19 *P* = 0.007). All data are shown as mean ± SEM. Statistics: one way-ANOVA with Tukey’s post hoc test (**b**), repeated measures ANOVA with Geisser-greenhouse correction and Tukey's post hoc test (**d-i**), or one-way ANOVA with Holm-Sidak (**m**–**o**). *P < 0.05, **P < 0.01, ***P < 0.001, ****P < 0.0001. *P*-values can be found in Additional file 5

We then assessed whether the observed changes in synaptic gene expression and synaptic engulfment by microglia upon deletion of CK2α′ were translated into enhanced synaptic density and neuronal activity in PS19 mice. We first conducted NeuN immunostaining analyses in the hippocampus of PS19 and PS19;CK2α′^(+/−)^ mice to determine if depletion of CK2α′ could impact the overall neuronal loss characteristic of PS19 mice [39, 89–91]. Significant depletion of NeuN^+^ cells and hippocampal atrophy were only seen in PS19 groups at a symptomatic age (Figs. 7a, b, S7a–c). Importantly, PS19;CK2α′^(+/−)^ mice showed a significant increase in the number of NeuN^+^ cells compared to PS19 in the CA1 (Figs. 7a, b, S7a–c).

We then evaluated if total synapse density was also positively altered in the CA1. We conducted immunostaining and colocalization analyses for VGlut1 (a pre-synaptic marker of excitatory synapses), and the postsynaptic marker PSD-95 (Fig. 7c–i). As expected, PS19 mice showed a significant decrease in the number of synapses (colocalized puncta VGlut1-PSD-95) compared to WT mice (Fig. 7f, i). The reduction in colocalized puncta in PS19 mice was due to a concomitant reduction in both PSD-95 and VGlut1 (Fig. 7d, e, g, h). In PS19;CK2α′^(+/−)^ mice, we observed a decrease in the % loss of both PSD-95 (Fig. 7d, g) and VGlut1 (Fig. 7e, h). This resulted in the amelioration of total synapse loss in PS19;CK2α′^(+/−)^ mice, with the level of colocalization comparable to that of WT (Fig. 7f, i). These results demonstrate a partial rescue of the density of excitatory synapses in the CA1 of PS19;CK2α′^(+/−)^ mice, aligning with the amelioration of hippocampal atrophy observed in this region (Fig. 7b) and the reduced synapse engulfment mediated by microglia (Fig. 6i, j). Consistently, silencing CK2α′ in primary cortical neuron cultures transfected with Tau-P301L-EGFP increased spine density compared to scramble (Fig. S8).

To investigate functional implications, we assessed hippocampal synaptic plasticity by performing extracellular fEPSP recordings in the CA1 region of acute hippocampal slices (Fig. 7j, k). TBS at the Schaffer collaterals induced STP, which resulted in a significant increase in synaptic efficacy at CA1 synapses in both WT and CK2α′^(+/−)^ mice, as evidenced by the enhanced fEPSP amplitude (170 ± 13%, *n* = 6; 170 ± 12%, *n* = 7) (Fig. 7l, m). In contrast, the increase in fEPSP amplitude was significantly reduced in PS19 and PS19;CK2α′^(+/−)^ mice compared to WT controls post TBS (123 ± 4%, *n* = 7; 128 ± 7%, *n* = 6; Fig. 7l, m), indicating impaired STP in these models.

Furthermore, we examined LTP at CA3-CA1 synapses, a form of synaptic plasticity linked to learning and memory. It has been previously shown that PS19 mice present impaired LTP in the CA1-CA3 pathway [39, 92, 93]. First, we observed significant differences in the input–output curves at CA3-CA1 synapses in PS19 and PS19;CK2α′^(+/−)^ mice when compared to WT, which suggests that the excitatory synaptic input properties were altered in these models (Fig. 7n). TBS at the Schaffer collaterals induced robust LTP in both WT (173 ± 12%, *n* = 6) and CK2α′^(+/−)^ (164 ± 9%, *n* = 7; Fig. 7l, o). However, in the PS19 mouse model, the magnitude of LTP was significantly diminished compared to WT (128 ± 9%, *n* = 7; *P* = 0.007 vs. WT) (Fig. 7l, o), suggesting a disruption in the molecular mechanisms underlying LTP. Importantly, in the PS19;CK2α′^(+/−)^ mice, LTP was partially restored (150 ± 6%, *n* = 6; *P* = 0.199 vs. WT) (Fig. 7l, o), pointing to a potential compensatory mechanism that partially mitigates the LTP deficits observed in the PS19 mice. Importantly, paired-pulse facilitation was unaltered across all experimental groups, suggesting that the observed changes in synaptic plasticity were most likely due to postsynaptic mechanisms dependent on the hippocampus (Fig. S9).

### CK2α′ haploinsufficiency improves PS19 behavior on Barnes maze

**Fig. 8:**
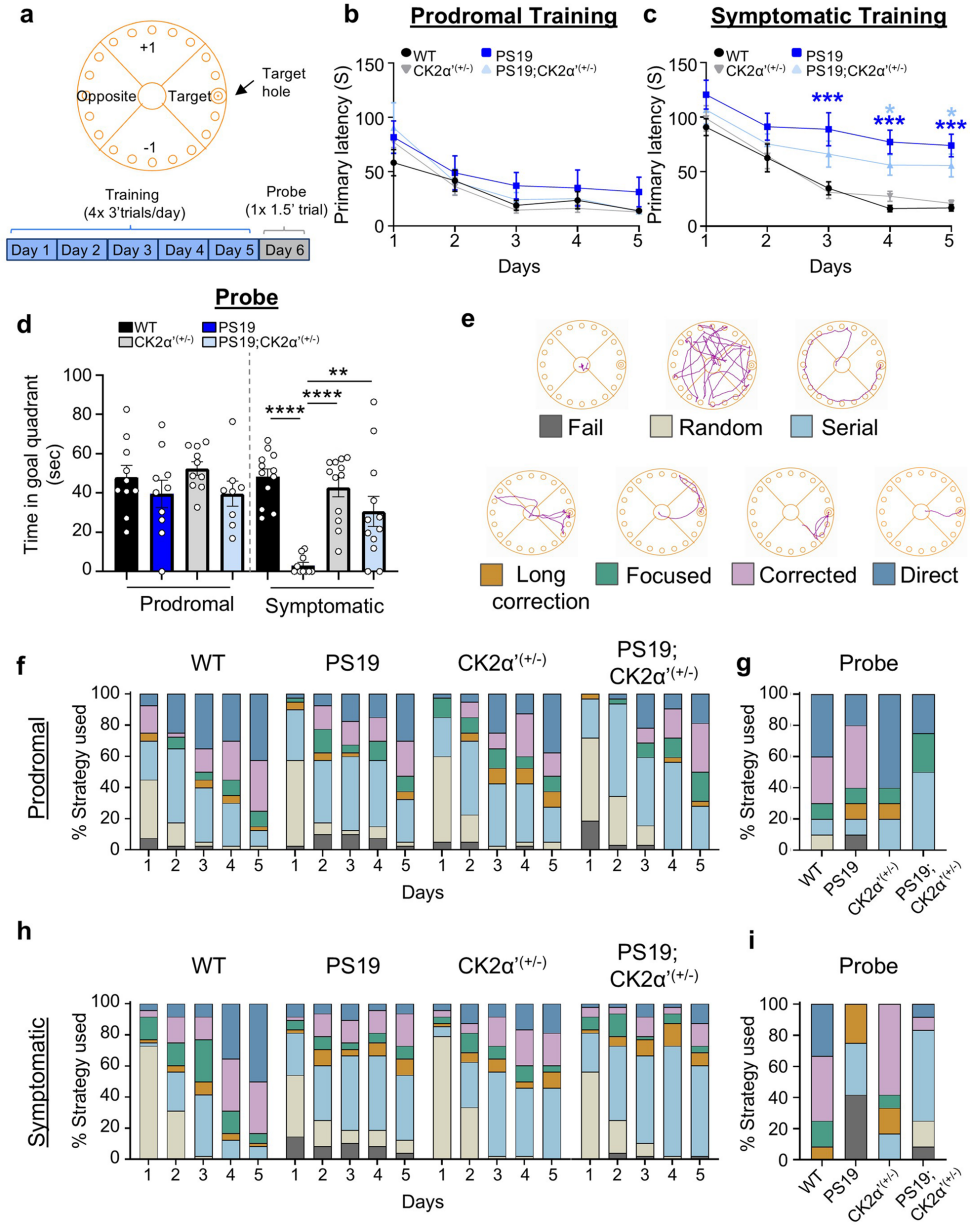
Depletion of CK2α′ improves spatial memory of PS19 mice on the Barnes maze. **a** Schematic of the Barnes maze divided into 4 quadrants (Target, Opposite, +1 and −1) and experimental set up. **b** and **c** Primary latency (s) to the escape hole at prodromal and symptomatic time points. Prodromal; genotype *P* = 0.0573, training day *P* < 0.0001 and interaction *P* = 0.9690. Symptomatic: genotype *P* < 0.0001, training day *P* < 0.0001 and interaction* P* = 0.7018. **d** Time spent in the goal quadrant during the probe trial at prodromal and symptomatic time points. *n* = 10–12 mice/genotype, 2 outliers were removed from the PS19 group using ROUT (Q = 1%). **e** Representative search strategy traces based on their navigation patterns on the maze. **f** and **h** Spatial strategies used to locate the escape hole across training days for each genotype at prodromal and symptomatic stages, respectively. **g** and **i** Spatial strategies used to locate the previously learned escape target hole during the probe trial. Data are shown as mean ± SEM. Two-way ANOVA with Bonferroni’s multiple comparisons (**b**, **c**) or One-Way ANOVA with Tukey’s post hoc test (**d**), *P < 0.05, **P < 0.01, ***P < 0.001, ****P < 0.0001. *P*-values can be found in Additional file 5

Finally, we assessed whether the overall amelioration in tau pathology, microgliosis and synaptic dysfunction mediated by CK2α′ had an impact on cognitive behaviors. Mice were trained in the Barnes maze task for 5 consecutive days on the location of a target hole (training) and on day 6 a probe trial was conducted with the target hole covered (Fig. 8a). During the training trials the primary latency to first locate the escape hole was recorded (Fig. 8b, c). All mice in the prodromal group demonstrated an improvement in the latency to locate the escape hole on day 1 vs day 5 (*P* = 0.0272 for WT; 0.0067 for PS19; 0.0002 for CK2α′^(+/−)^; *P* < 0.0001 for PS19;CK2α′^(+/−)^) with no significant difference between the genotypes on any individual day (Fig. 8b). All mice in the symptomatic group also demonstrated a significant improvement on day 1 versus day 5 (*P* < 0.0001 for WT; *P* = 0.0109 for PS19; *P* < 0.0001 for CK2α′^(+/−)^; *P* = 0.0037 for PS19;CK2α′^(+/−)^). However, PS19 and PS19;CK2α′^(+/−)^ showed a significant impairment compared to WT, starting on day 3 for PS19 (*P* = 0.0009) and day 4 for PS19;CK2α′^(+/−)^ (*P* = 0.0306) (Fig. 8c). The training data suggest that both symptomatic PS19 and PS19;CK2α′^(+/−)^ mice showed impaired learning on the Barnes maze compared to WT mice, although the deficits in PS19;CK2α′^(+/−)^ were attenuated.

Following the training phase of the Barnes maze, we conducted a probe phase in which the escape hole was covered. At the prodromal age, there was no significant difference in the time spent in the escape hole quadrant for any genotype (Fig. 8d). At the symptomatic age, the PS19 mice spent significantly decreased time in the goal quadrant compared to WT (*P* < 0.0001) (Fig. 8d). Importantly, the PS19;CK2α′^(+/−)^ mice spent significantly more time than the PS19 mice in the goal quadrant with no significant difference from WT (*P* = 0.0038 vs. PS19, *P* = 0.0804 vs. WT), demonstrating a rescue of spatial memory of the PS19;CK2α′^(+/−)^ mice (Fig. 8d).

The PS19 mouse model is known to present motor deficits due to hindlimb paralysis and weakness [39], which could confound Barnes maze results. The prodromal mice displayed no difference in the distance traveled on Barnes maze (Fig. S10a). While the symptomatic PS19 mice traveled a less distance in the Barnes maze compared to WT mice, they did not show a significant difference in the distance traveled compared to PS19;CK2α′^(+/−)^ mice (Fig. S10b). In the open-field testing, we found no differences between groups at the prodromal age (Fig. S10c, d). On the contrary, the symptomatic mice showed significant hyperactivity in the open field task, demonstrating the ability of PS19 mice to move (Fig. S10e, f). Indeed, no significant differences in hyperactivity were observed between PS19 and PS19;CK2α′^(+/−)^ mice (Fig. S10e). This finding supports that the improvement in cognitive abilities seen in the PS19;CK2α′^(+/−)^ mice is not due to changes in motor behavior but rather is related to an improvement in memory.

Mice can use different cognitive strategies to navigate the Barnes maze and locate the escape hole [55]. As the mice learn the task they show improvement in the strategy used to locate the escape hole [55] (Fig. 8e). Assessing these cognitive strategies may provide a deeper insight into how memory and spatial learning are being utilized. All animals in the prodromal group demonstrated a similar pattern in spatial strategies over the training phase (Fig. 8f) and showed a predominance in the corrected and direct strategies in the probe phase (Fig. 8g). Symptomatic PS19 and PS19;CK2α′^(+/−)^ mice both showed impaired spatial strategies over the training phase. By day 5, both genotypes largely displayed either failing, random or serial strategies (Fig. 8h). However, during the shortened probe phase, PS19 displayed a worsened overall strategy, with predominant failing, serial, and long-correction strategies compared to PS19;CK2α′^(+/−)^ mice that displayed more serial, corrected and direct strategies (Fig. 8i). Overall, the PS19;CK2α′^(+/−)^ mice displayed improvement in the probe phase of the Barnes maze, in both the time spent in the escape quadrant and the strategy used to locate the target, indicating improvements in spatial memory.

## Discussion

Tauopathies are a heterogeneous group of neurodegenerative disorders for which effective treatments are still not available, in part due to an incomplete understanding of the underlying mechanisms. In this study, we present a novel target, CK2α′, a catalytic subunit of CK2, as a potential upstream regulator of tau-mediated neurodegeneration, acting at the intersection of neuroimmune signaling and synaptic dysfunction.

Here, we report that CK2α′ expression is abnormally elevated in the brains of dementia patients and in the hippocampus of PS19 mice, with preferential upregulation observed in hippocampal neurons and microglia. These findings are supported by multiple single-cell RNA-sequencing (snRNA-seq) studies in human AD, which consistently show increased CK2α′ expression in excitatory neurons [69–71]. Additionally, the study by Gerrits et al. [68] in the occipital lobe (OL) has reported preferential CK2α′ expression in AD-associated microglia. Other scRNA-seq studies have detected elevated CK2α′ expression in various brain cell types, with patterns varying by dataset and brain region [69–71]. On the other hand, CK2α seems to be increased in cortical astrocytes of the 5×FAD and PS19 models [25]. This collective evidence suggests that CK2α′ dysregulation in AD/ADRD may be both cell-type- and region-specific. Consistent with our findings, a concurrently submitted study has reported that CK2α′ is elevated in AD models and contributes to APP-regulated glial inflammation [94], independently supporting and strengthening our conclusions.

Increased CK2 levels have been associated with tau pathology [17, 18, 24, 95], microglial state [96, 97] and neuroinflammation more broadly [98–104]. However, many of these studies were performed in cell-based systems and/or in vivo using pan-CK2 inhibitors or ectopic overexpression of CK2 subunits [17, 18, 24, 35]. As a result, the specific contributions of individual CK2 catalytic subunits to tau pathology have remained largely unresolved. To our knowledge, no prior studies have genetically targeted CK2α or CK2α′ in tauopathy using a comparable loss-of-function approach to that employed here, making this study the first to directly interrogate CK2α′ function in this context.

Our studies in vitro using N2A cells transfected with Tau-P301L showed that silencing CK2α′, but not CK2α, decreased pTau accumulation. We therefore decided to explore the impact of reducing the levels of CK2α′ in vivo. We used CK2α′ heterozygous null mice because our goal was to restore CK2α′ to physiological levels in PS19 mice and avoid any potential off target effects due to complete deletion of CK2α′, for which a heterozygous approach is more appropriate than complete knockout. Moreover, partial reduction of CK2α′ has proven to be efficacious in ameliorating HD-like symptoms in an HD mouse model [14, 15], and has greater translational relevance, as any future pharmacological inhibition is unlikely to achieve complete enzyme suppression. Our studies in vivo reducing the levels of CK2α′ by ~50% in the PS19 model influenced tau pathology by decreasing the accumulation of pTau and tau burden in the hippocampus and cortex of PS19;CK2α′^(+/−)^ mice. In support to our data, previous studies in vitro showed that overexpression of CK2α′ subunit decreased the activity of I2PP2A/SET, a cellular protein that functions as an endogenous inhibitor of protein phosphatase 2A (PP2A), increasing pTau. In contrast, overexpression of CK2α increased the activity of I2PPA2a/SET [95]. Although early studies demonstrated that tau is a physiological substrate of CK2, at least in specific contexts such as neurogenesis [105], the findings by Perez et al. [95] suggest that the effects we observed after manipulating CK2α′ on pTau accumulation could be mediated through intermediary molecules that modulate tau phosphorylation.

Haploinsufficiency of CK2α′ also increased the expression of synaptic genes, reflected by transcriptional changes in genes involved in synaptic organization and vesicular transport (WGCNA module #3), excitatory synaptic density, and synaptic function via LTP in the PS19 mice. Along these lines, haploinsufficiency of CK2α′ in a mouse model of HD also showed improved synaptic density and function [15, 52], although how exactly CK2α′ influences these processes is still unknown. A previous study showed that CK2α′ can influence the expression of synaptic genes, especially those related to glutamatergic excitatory signaling [15]. A more recent study showed that pharmacological inhibition of CK2 mitigated AD tau pathology by preventing synaptic mislocalization of the NMDA receptor subunit NR2B [24]. NR2B mediates long-term depression (LTD), and LTD specifically induces the phosphorylation of tau. These studies support our findings and strengthen the relationship between CK2α′, tau phosphorylation, and the regulation of neuronal function in both AD and other neurodegenerative diseases. Importantly, the timing of this relationship warrants further investigation. Although CK2α′ is expressed at relatively stable levels in the human brain throughout development to adulthood, as reported by the Allen Brain Atlas Brain Span database [106], it is possible that deletion of CK2α′ from embryo in PS19 causes its effects through developmental or early alterations that manifest later in life. To address this question, future inducible deletions of CK2α′ in adult mice are necessary to determine the timing at which CK2α′ intervention is needed to ameliorate tau pathology. In addition, since CK2α′ is found elevated in both neurons and microglia, it is still unknown whether the effects mediated by CK2α′ in pTau accumulation and synaptic function are cell or non-cell autonomous.

CK2 has been identified as a mediator of microglial reactivity and inflammation in different models [96, 97] and it has been well-established as a mediator of inflammation in a variety of contexts including SARS-CoV infection, cancers, bacterial infections, intestinal inflammation and renal failure [98–103]. This is largely mediated via CK2’s involvement in regulating several large inflammatory factors such as NF-κB, STAT1, and EGR-1 [104]. It is known that chronic activation of NF-kB increases the accumulation of pTau [9]. On the other hand, pathological tau has also been shown to induce inflammation and NF-kB activation by interacting directly with inflammatory receptors such as toll-like receptors 2 [10, 11], creating a vicious cycle of worsening inflammation and tau pathology [9–12]. Our data demonstrated that CK2α′ haploinsufficiency significantly reduced the number of Iba1^+^ cells in the hippocampus, reduced the levels of the microglial reactive and phagocytic markers CD68 and Clec7a, ameliorated microglia morphological changes associated with pathology, reduced the levels of pro-inflammatory cytokines and reduced microglia-mediated synaptic engulfment in PS19 mice. In support to our findings, previous studies in human microglia derived from human induced pluripotent stem cells (hiPSCs) treated with CK2 inhibitors also reduced cytokine production [97]. Therefore, all together, these data support a specific key role of CK2α′ in mediating neuroinflammation via activation of microglia.

An important question that remains to be answered is whether microglial-CK2α′-mediated changes in microglia phenotypes and neuroinflammation can directly impact pTau accumulation. Recent literature supports the notion that inflammatory cytokines and chemokines released by microglia can promote pTau [9, 12, 107–109]. Our data obtained in vitro using N2A cells transfected with hTau-P301L-EGFP would suggest that the impact of CK2α′ on pTau may be neuronal dependent since no microglia were present in these cultures. However, it is still possible that microglial-CK2α′ influences neuronal pTau proteostasis in vivo by mediating changes in microglia reactivity and cytokines production. Our RNA-seq and WGCNA also revealed an important dysregulated module in PS19 mice (module #3) that was further upregulated in PS19;CK2α′^(+/−)^ animals and was enriched in microglial-related genes involved in stress response and neuroimmune signaling. At first glance, this could be interpreted as an exacerbation of neuroinflammation in PS19;CK2α′^(+/−)^ animals. However, our complementary morphological and phenotypic analyses demonstrate improved microglial profiles and reduced inflammatory cytokine production in PS19;CK2α′^(+/−)^ mice. Taken together, these findings suggest that the transcriptional changes observed by RNA-seq reflect a more effective and coordinated neuroimmune response rather than a detrimental one. In this context, the elevated neuroimmune gene expression in PS19 mice likely represents a response to the presence of hTau that is insufficient to confer protection. In contrast, the further enhancement observed in PS19;CK2α′(+/−) mice appeared to correspond to a more functional, potentially protective immune response capable of better counteracting the accumulation of pTau. Importantly, our RNA-seq analyses were performed at a prodromal stage, and the transcriptional changes observed in the tan and turquoise modules preceded any detectable differences in pTau levels. This temporal relationship suggests that early activation of neuroimmune pathways, including microglial responses, may prime downstream processes that ultimately influence neuronal stages and pTau proteostasis.

Loss of synaptic density and neuronal dysfunction in AD models have also been previously connected to increased phagocytic microglia [64, 83, 110, 111]. Our results demonstrated a decrease in microglia phagocytosis of the PSD-95 synaptic marker in PS19;CK2α′^(+/–)^ mice, suggesting that CK2α′ is influencing the phagocytosis of synapses by microglia, but the mechanisms behind this are yet to be elucidated. CK2α′ could facilitate these processes by regulating the transcription of genes involved in phagocytosis and synaptic engulfment and/or by phosphorylating and activating specific proteins related to these processes. Deletion of CK2α′ in PS19 mice resulted in the down-regulation of several genes related to apoptosis/phagocytosis and synapse engulfment such as *Cept1, Ube2n, Oxct1, Exoc3, Adgrb1* and *Sh3glb2 *[75, 76, 112–116] which can contribute to the phenotypes observed in PS19;CK2α′^(+/–)^ mice. It is also possible that CK2α′ phosphorylates elements of the complement component such as C9 and C1r, known CK2 substrates [117, 118], and influence microglia-mediated synaptic pruning.

Importantly, CK2α′ haploinsufficiency improved memory functions of PS19 symptomatic mice assessed in the Barnes maze. In support of our results, previous studies overexpressing CK2 subunits in WT or AD mice worsened cognitive functions [18] while treatment with CK2 inhibitors ameliorated cognitive dysfunction in models of AD [25]. We recognize that motor deficits in PS19 mice such hindlimb paralysis and weakness [39] and/or hyperactivity could confound our interpretation of improved memory based on Barnes Maze results. However, although the symptomatic PS19 mice traveled a less distance in the Barnes Maze compared to WT mice, they did not show a significant difference in distance traveled compared to PS19;CK2α′^(+/−)^ mice. PS19 mice also presented a characteristic hyperactive phenotype in open-field testing, demonstrating their ability to move, and such phenotype was not significantly different from PS19;CK2α′^(+/−)^ mice. Altogether, the data support our conclusion that upregulation of CK2α′ in the brain of PS19 mice contributes to cognitive alterations and memory dysfunction.

In conclusion, our findings identified CK2α′ as a central and previously underappreciated regulator of tauopathy pathogenesis, acting at the crossroads of neuroimmune signaling, synaptic function, and tau phosphorylation. By demonstrating that CK2α′ haploinsufficiency alleviates tau pathology, reduces neuroinflammation, and improves synaptic integrity and function in PS19 mice, we provide compelling genetic evidence that CK2α′ contributes to multiple pathological hallmarks of tau-driven neurodegeneration. Importantly, these effects appear to occur through complex, context-dependent mechanisms that may differ across cell types and disease stages. The observation that CK2α′ influences both neuronal and microglial compartments underscores the need to dissect its cell-autonomous versus non-cell-autonomous roles. Given the growing interest in CK2 as a therapeutic target and the limitations of current inhibitors that lack subunit specificity, the development of CK2α′-selective modulators [94, 119] may represent a promising avenue for disease-modifying therapies in tauopathies and related neurodegenerative conditions. Future studies elucidating the molecular and cellular pathways downstream of CK2α′ will be critical for translating these findings into clinical applications.

## Conclusion

The presented work demonstrates a role of CK2α′ in modifying outcomes related to microglia reactivity, phagocytosis and synaptic function in tauopathy. These results highlight CK2α′ as a potential therapeutic target, where inhibition could have beneficial effects in disease progression.

## Supplementary Information

Additional file 1.  **Figure S1**. Technical validation of CK2α’ detection methods and extended tau correlation analyses. **Figure S2**. CSNK2a2 scRNA-seq expression in human brain tissue. **Figure S3**. PS19;CK2α’^(+/-)^ mice display decreased AT8 staining in the cortex and AT100 in hippocampus of symptomatic mice. **Figure S4**. Gene Ontology Term:Biological Processes (BP) for top biological pathways for the 11 the significant WGCNA modules. **Figure S5**. CK2α’ haploinsufficency decreased Iba1^+^ microglia cell number in the hippocampus and overlaying cortex of PS19 mice. **Figure S6**. CK2α’ haploinsufficency decreased GFAP^+^ astrocytes in the overlaying cortex but not in the hippocampus of PS19 mice. **Figure S7**. Decrease NeuN^+^ cells in the CA3 and DG of both PS19 and PS19;CK2α’^(+/-)^ mice. **Figure S8**. Silencing CK2α’ in primary cortical cells expressing Tau-P301L increases dendritic spine density. **Figure S9**. Paired pulse ratio of hippocampal slices shows no deficits between genotypes. **Figure S10**. Symptomatic PS19 demonstrates decreased movement on Barnes Maze, but hyperactive phenotype in open field.

Additional file 2. NBB Human sample information.

Additional file 3. RNA-Seq module information.

Additional file 4. Uncropped Blots.

Additional file 5. List of *P*-values for Figs. 1–8 and Supplementary Figures.

## Data Availability

RNA-Seq data generated in this study are available in the Gene Expression Omnibus repository found at https://www.ncbi.nlm.nih.gov/geo/ with accession number GSE298505. The reviewer token to access the GEO deposited data is mlwxmuactlibryl. Allan Brain Atlas Aging, Dementia and TBI dataset that supports conclusions of this article is publicly available at https://aging.brain-map.org/overview/home [60]. Human brain tissue was received from the NIH NeuroBioBank repository and is available publicly for request at https://neurobiobank.nih.gov/. scRNA-Seq study data from human samples that contributed to conclusions of this article can be found in The Alzheimer's Cell Atlas (TACA) https://taca.lerner.ccf.org/ [67–71].

## Abbreviations

AD: Alzheimer's disease
ADRD: Alzheimer's disease related dementias
APP: Amyloid precursor protein
DEG: Differentially expressed gene
fEPSP: Field excitatory postsynaptic potential
FTD: Frontotemporal dementia
GO: Gene ontology
HD: Huntington’s disease
hIPSCs: Human induced pluripotent stem cells
LTP: Long-term potentiation
PP2A: Protein phosphatase 2
pTau: Phosphorylated tau
snRNA-seq: Single cell Rna-sequencing
STP: Short term potentiation
TACA: The Alzheimer’s Cell Atlas
TBS: Theta burst stimulation
WGCNA: Weighted gene co-expression network analysis
