# Supplementary material for: Protein kinase CK2α′ as a dual modulator of neuroimmune signaling and synaptic dysfunction in tauopathy
Source: Transl Neurodegener. 2026 Jul 9;15:31. doi: 10.1186/s40035-026-00563-3 (PMC13348822; doi:10.1186/s40035-026-00563-3)
Supplement: Supplementary file 1 — Additional file 1. Figure S1. Technical validation of CK2α’ detection methods and extended tau correlation analyses. Figure S2. CSNK2a2 scRNA-seq expression in human brain tissue. Figure S3. PS19;CK2α’(+/-) mice display decreased AT8 staining in the cortex and AT100 in hippocampus of symptomatic mice. Figure S4. Gene Ontology Term:Biological Processes (BP) for top biological pathways for the 11 the significant WGCNA modules. Figure S5. CK2α’ haploinsufficency decreased Iba1+ microglia cell number in the hippocampus and overlaying cortex of PS19 mice. Figure S6. CK2α’ haploinsufficency decreased GFAP+ astrocytes in the overlaying cortex but not in the hippocampus of PS19 mice. Figure S7. Decrease NeuN+ cells in the CA3 and DG of both PS19 and PS19;CK2α’(+/-) mice. Figure S8. Silencing CK2α’ in primary cortical cells expressing Tau-P301L increases dendritic spine density. Figure S9. Paired pulse ratio of hippocampal slices shows no deficits between genotypes. Figure S10. Symptomatic PS19 demonstrates decreased movement on Barnes Maze, but hyperactive phenotype in open field. [file 40035_2026_563_MOESM1_ESM.pptx]

## Slide 1
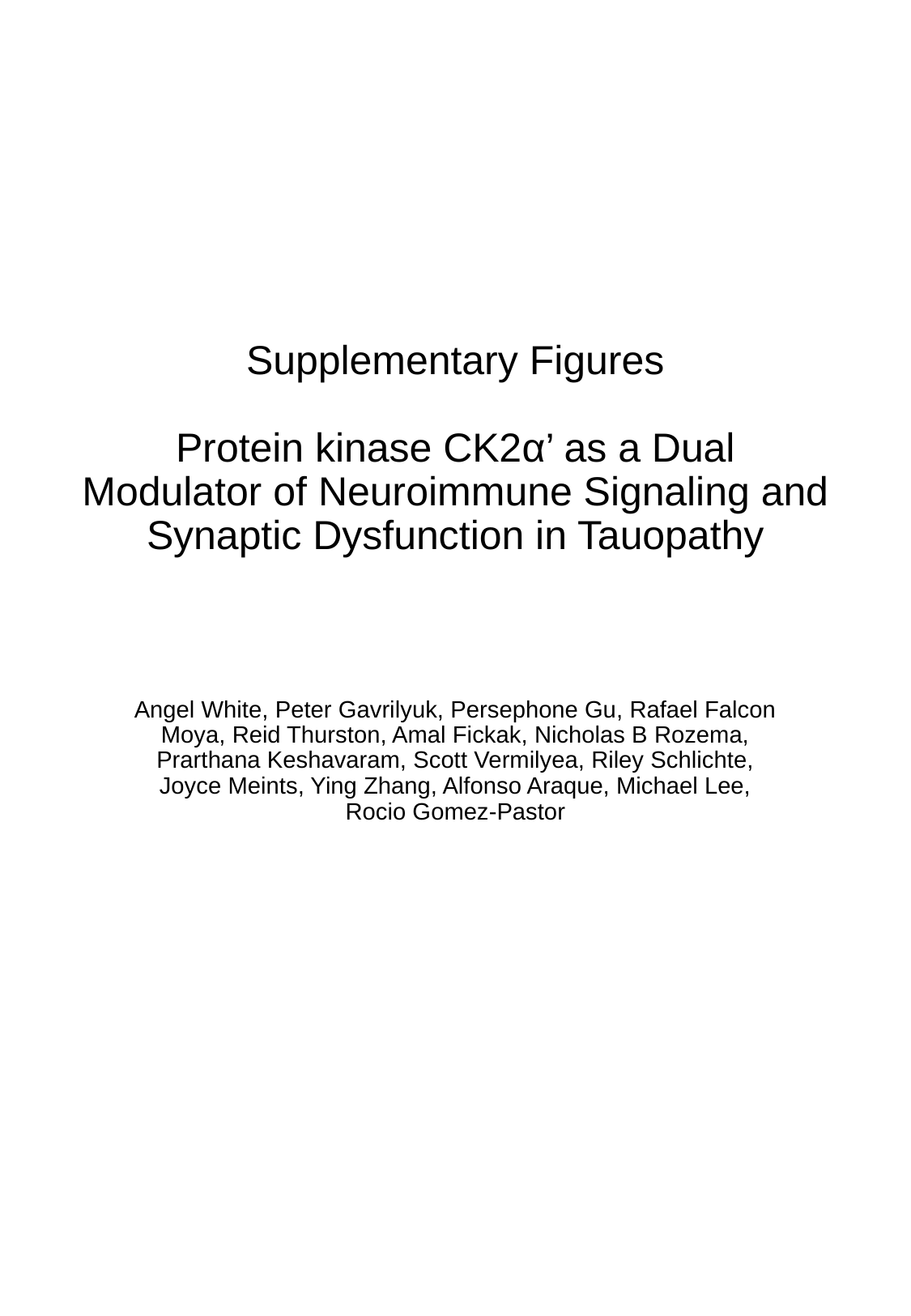

# Supplementary Figures Protein kinase CK2α’ as a Dual Modulator of Neuroimmune Signaling and Synaptic Dysfunction in Tauopathy
Angel White, Peter Gavrilyuk, Persephone Gu, Rafael Falcon Moya, Reid Thurston, Amal Fickak, Nicholas B Rozema, Prarthana Keshavaram, Scott Vermilyea, Riley Schlichte, Joyce Meints, Ying Zhang, Alfonso Araque, Michael Lee, Rocio Gomez-Pastor

## Slide 2
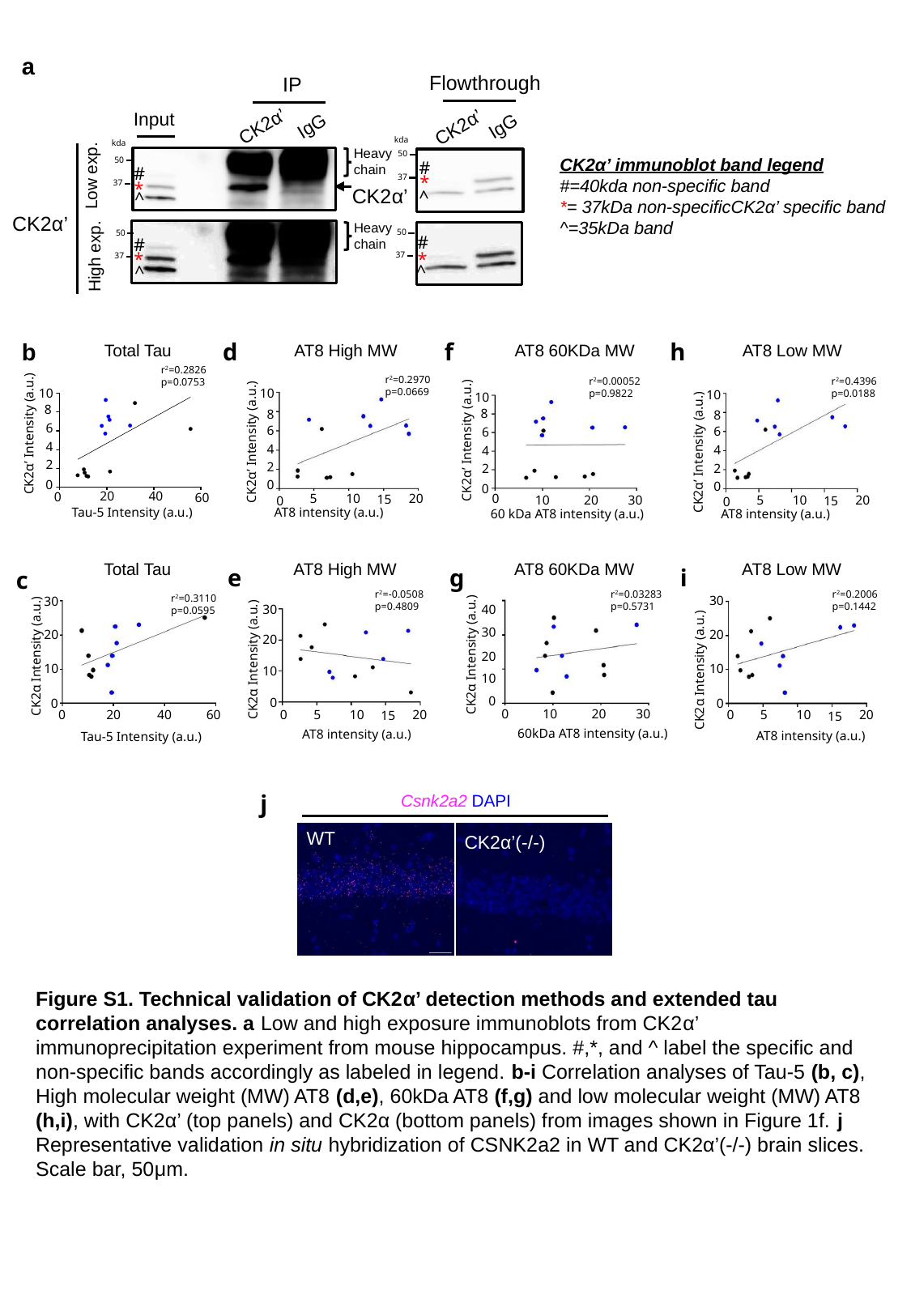

a
Flowthrough
IP
IgG
IgG
CK2α’
Input
CK2α’
kda
kda
Heavy
chain
50
CK2α’ immunoblot band legend
#=40kda non-specific band
*= 37kDa non-specificCK2α’ specific band
^=35kDa band
50
#
#
Low exp.
37
*
37
*
CK2α’
^
^
CK2α’
Heavy
chain
50
50
#
#
High exp.
*
*
37
37
^
^
b
d
f
h
Total Tau
AT8 High MW
AT8 60KDa MW
AT8 Low MW
10
8
6
4
2
0
CK2α’ Intensity (a.u.)
0
10
20
30
60 kDa AT8 intensity (a.u.)
r2=0.2826
p=0.0753
r2=0.2970
p=0.0669
r2=0.4396
p=0.0188
r2=0.00052
p=0.9822
10
8
6
4
2
0
10
10
8
6
4
2
0
20
5
10
15
0
AT8 intensity (a.u.)
8
6
CK2α’ Intensity (a.u.)
CK2α’ Intensity (a.u.)
4
CK2α’ Intensity (a.u.)
2
0
20
40
0
60
20
5
10
15
0
Tau-5 Intensity (a.u.)
AT8 intensity (a.u.)
e
g
i
Total Tau
AT8 High MW
AT8 60KDa MW
AT8 Low MW
c
40
30
CK2α Intensity (a.u.)
20
10
0
0
10
20
30
60kDa AT8 intensity (a.u.)
r2=-0.0508
p=0.4809
r2=0.03283
p=0.5731
r2=0.2006
p=0.1442
r2=0.3110
p=0.0595
30
20
10
0
20
5
10
0
15
AT8 intensity (a.u.)
30
30
20
20
CK2α Intensity (a.u.)
CK2α Intensity (a.u.)
10
CK2α Intensity (a.u.)
10
0
0
0
20
40
60
20
5
10
0
15
AT8 intensity (a.u.)
Tau-5 Intensity (a.u.)
j
Csnk2a2 DAPI
WT
CK2α’(-/-)
Figure S1. Technical validation of CK2α’ detection methods and extended tau correlation analyses. a Low and high exposure immunoblots from CK2α’ immunoprecipitation experiment from mouse hippocampus. #,*, and ^ label the specific and non-specific bands accordingly as labeled in legend. b-i Correlation analyses of Tau-5 (b, c), High molecular weight (MW) AT8 (d,e), 60kDa AT8 (f,g) and low molecular weight (MW) AT8 (h,i), with CK2α’ (top panels) and CK2α (bottom panels) from images shown in Figure 1f. j Representative validation in situ hybridization of CSNK2a2 in WT and CK2α’(-/-) brain slices. Scale bar, 50μm.

## Slide 3
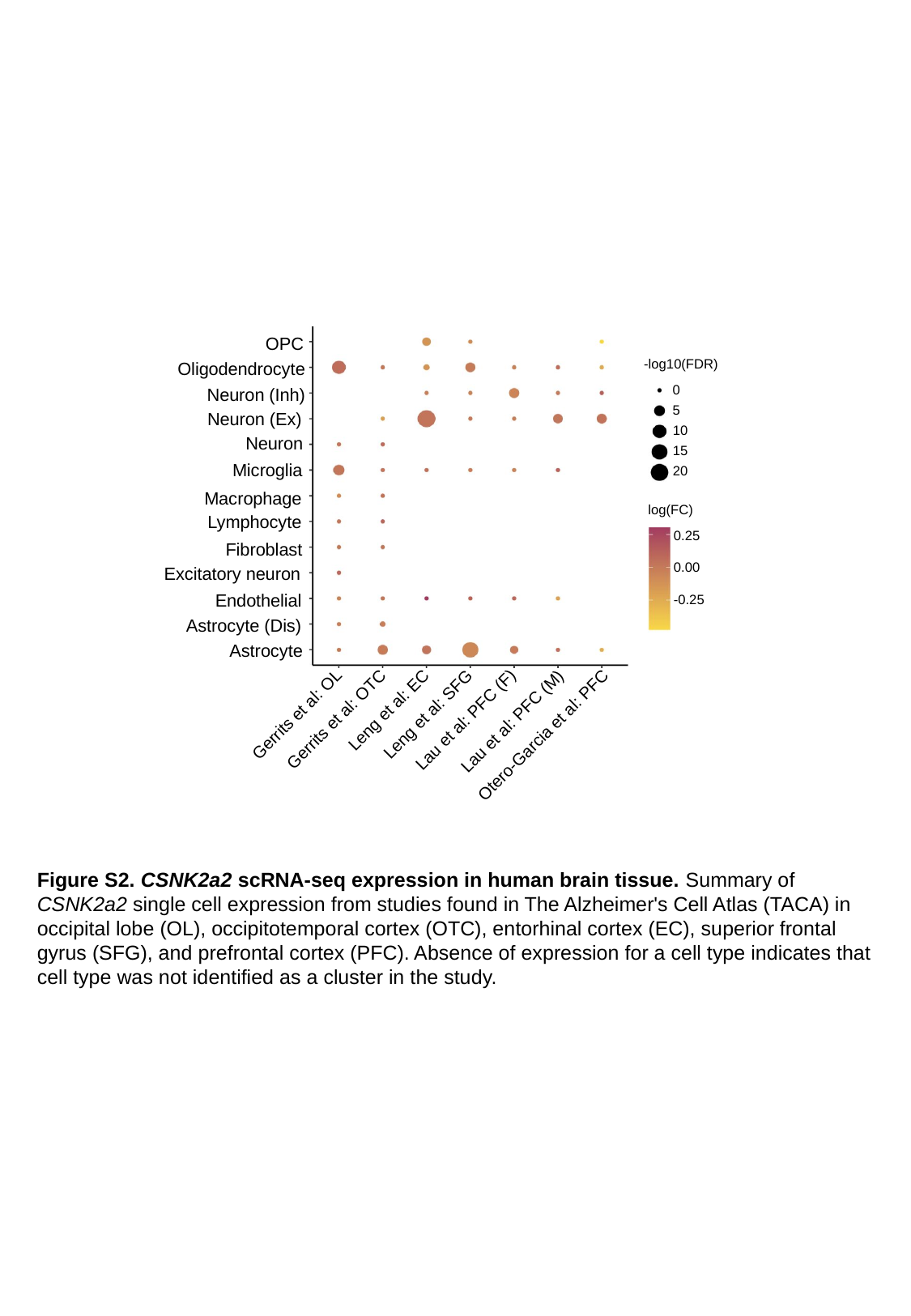

OPC
Oligodendrocyte
Neuron (Inh)
Neuron (Ex)
Neuron
Microglia
Macrophage
Lymphocyte
Fibroblast
Excitatory neuron
Endothelial
Astrocyte (Dis)
Astrocyte
-log10(FDR)
0
5
10
15
20
log(FC)
0.25
0.00
-0.25
Leng et al: EC
Leng et al: SFG
Gerrits et al: OL
Gerrits et al: OTC
Lau et al: PFC (F)
Lau et al: PFC (M)
Otero-Garcia et al: PFC
Figure S2. CSNK2a2 scRNA-seq expression in human brain tissue. Summary of CSNK2a2 single cell expression from studies found in The Alzheimer's Cell Atlas (TACA) in occipital lobe (OL), occipitotemporal cortex (OTC), entorhinal cortex (EC), superior frontal gyrus (SFG), and prefrontal cortex (PFC). Absence of expression for a cell type indicates that cell type was not identified as a cluster in the study.

## Slide 4
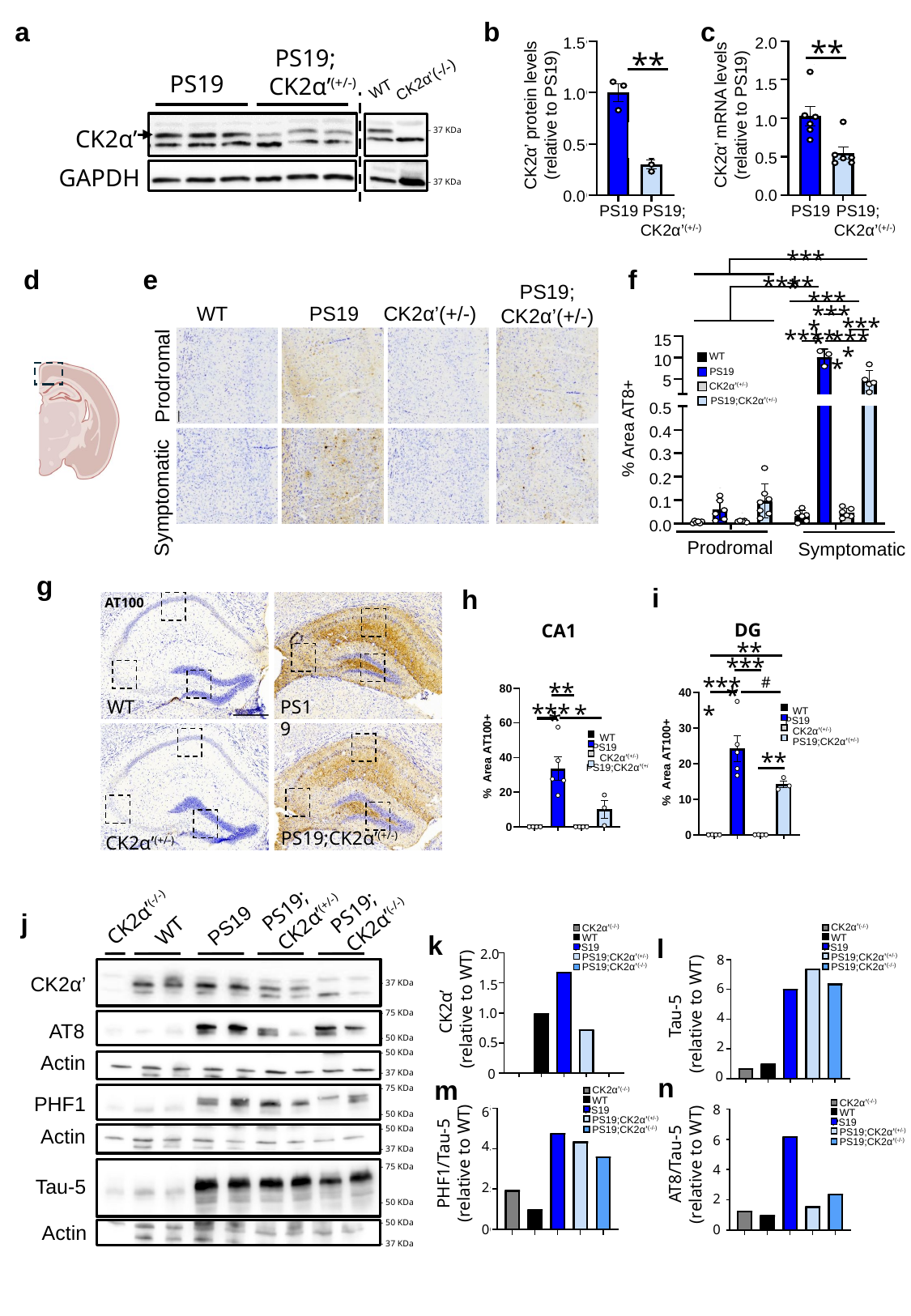

a
b
c
**
1.5
1.0
0.5
0.0
CK2α’ protein levels
(relative to PS19)
PS19
PS19;
 CK2α’(+/-)
2.0
1.5
1.0
0.5
0.0
CK2α’ mRNA levels
(relative to PS19)
PS19
PS19;
 CK2α’(+/-)
 PS19;
CK2α’(+/-)
PS19
CK2α’
GAPDH
CK2α’(-/-)
WT
**
a
- 37 KDa
- 37 KDa
PS19;
CK2α’(+/-)
CK2α’(+/-)
PS19
WT
Prodromal
Symptomatic
****
****
****
****
****
****
****
d
e
f
Prodromal
Symptomatic
15
10
5
0.5
0.4
0.3
0.2
0.1
0.0
% Area AT8+
WT
PS19
CK2α’(+/-)
PS19;CK2α’(+/-)
g
i
h
AT100
PS19
WT
PS19;CK2α’(+/-)
CK2α’(+/-)
DG
**
****
****
#
WT
PS19
CK2α’(+/-)
PS19;CK2α’(+/-)
**
CA1
***
***
*
WT
PS19
CK2α’(+/-)
PS19;CK2α’(+/-)
PS19;
CK2α’(+/-)
PS19;
CK2α’(-/-)
CK2α’(-/-)
PS19
WT
CK2α’
- 37 KDa
- 75 KDa
AT8
- 50 KDa
- 50 KDa
Actin
- 37 KDa
- 75 KDa
PHF1
- 50 KDa
Actin
- 50 KDa
- 37 KDa
- 75 KDa
Tau-5
- 50 KDa
- 50 KDa
Actin
- 37 KDa
j
CK2α’(-/-)
WT
PS19
PS19;CK2α’(+/-)
PS19;CK2α’(-/-)
CK2α’(-/-)
WT
PS19
PS19;CK2α’(+/-)
PS19;CK2α’(-/-)
k
l
2.0
8
1.5
6
CK2α’
(relative to WT)
Tau-5
(relative to WT)
1.0
4
0.5
2
n
0
m
0
CK2α’(-/-)
WT
PS19
PS19;CK2α’(+/-)
PS19;CK2α’(-/-)
CK2α’(-/-)
WT
PS19
PS19;CK2α’(+/-)
PS19;CK2α’(-/-)
8
6
6
4
PHF1/Tau-5
(relative to WT)
AT8/Tau-5
(relative to WT)
4
2
2
0
0

## Slide 5
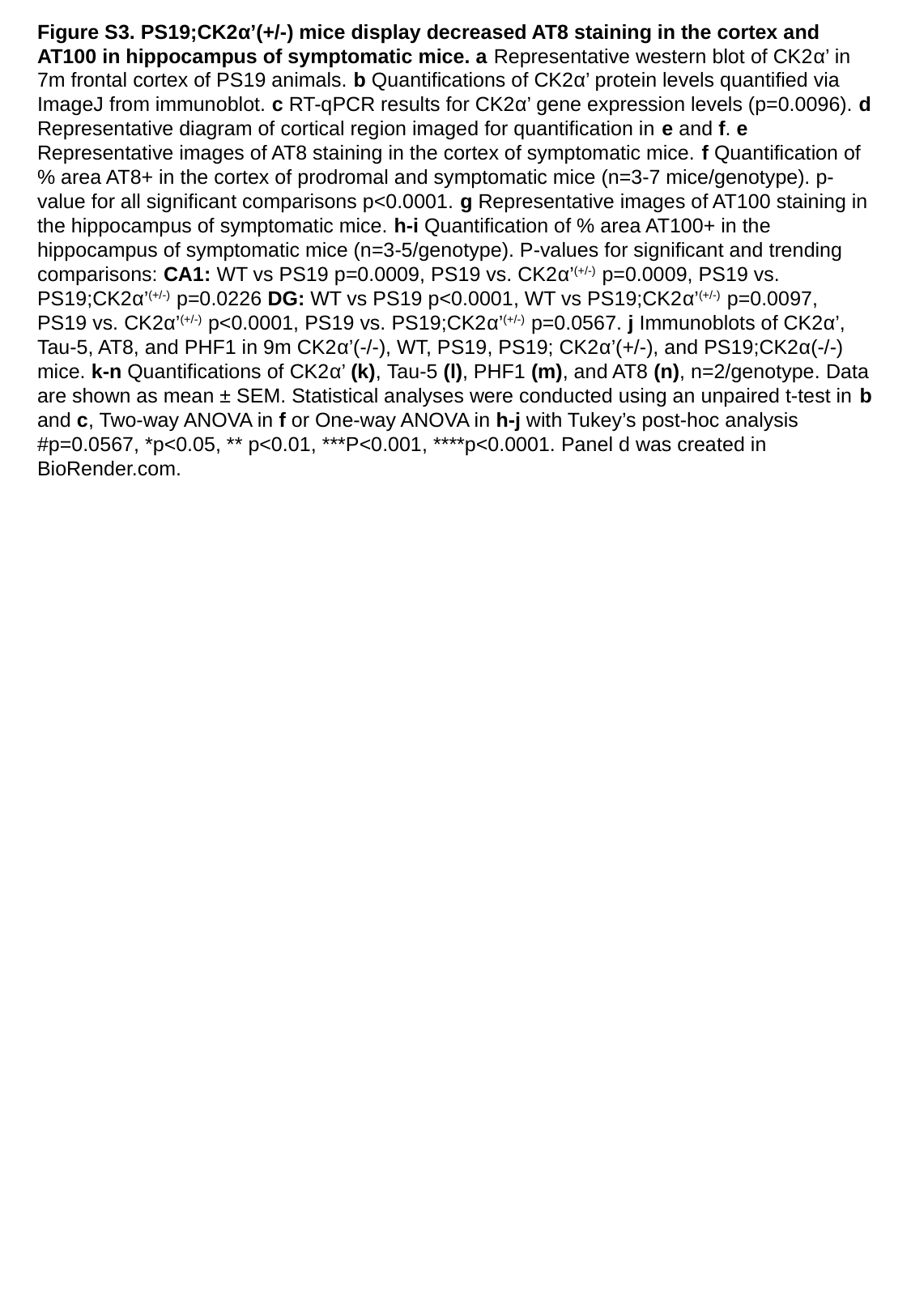

Figure S3. PS19;CK2α’(+/-) mice display decreased AT8 staining in the cortex and AT100 in hippocampus of symptomatic mice. a Representative western blot of CK2α’ in 7m frontal cortex of PS19 animals. b Quantifications of CK2α’ protein levels quantified via ImageJ from immunoblot. c RT-qPCR results for CK2α’ gene expression levels (p=0.0096). d Representative diagram of cortical region imaged for quantification in e and f. e Representative images of AT8 staining in the cortex of symptomatic mice. f Quantification of % area AT8+ in the cortex of prodromal and symptomatic mice (n=3-7 mice/genotype). p-value for all significant comparisons p<0.0001. g Representative images of AT100 staining in the hippocampus of symptomatic mice. h-i Quantification of % area AT100+ in the hippocampus of symptomatic mice (n=3-5/genotype). P-values for significant and trending comparisons: CA1: WT vs PS19 p=0.0009, PS19 vs. CK2α’(+/-) p=0.0009, PS19 vs. PS19;CK2α’(+/-) p=0.0226 DG: WT vs PS19 p<0.0001, WT vs PS19;CK2α’(+/-) p=0.0097, PS19 vs. CK2α’(+/-) p<0.0001, PS19 vs. PS19;CK2α’(+/-) p=0.0567. j Immunoblots of CK2α’, Tau-5, AT8, and PHF1 in 9m CK2α’(-/-), WT, PS19, PS19; CK2α’(+/-), and PS19;CK2α(-/-) mice. k-n Quantifications of CK2α’ (k), Tau-5 (l), PHF1 (m), and AT8 (n), n=2/genotype. Data are shown as mean ± SEM. Statistical analyses were conducted using an unpaired t-test in b and c, Two-way ANOVA in f or One-way ANOVA in h-j with Tukey’s post-hoc analysis #p=0.0567, *p<0.05, ** p<0.01, ***P<0.001, ****p<0.0001. Panel d was created in BioRender.com.

## Slide 6
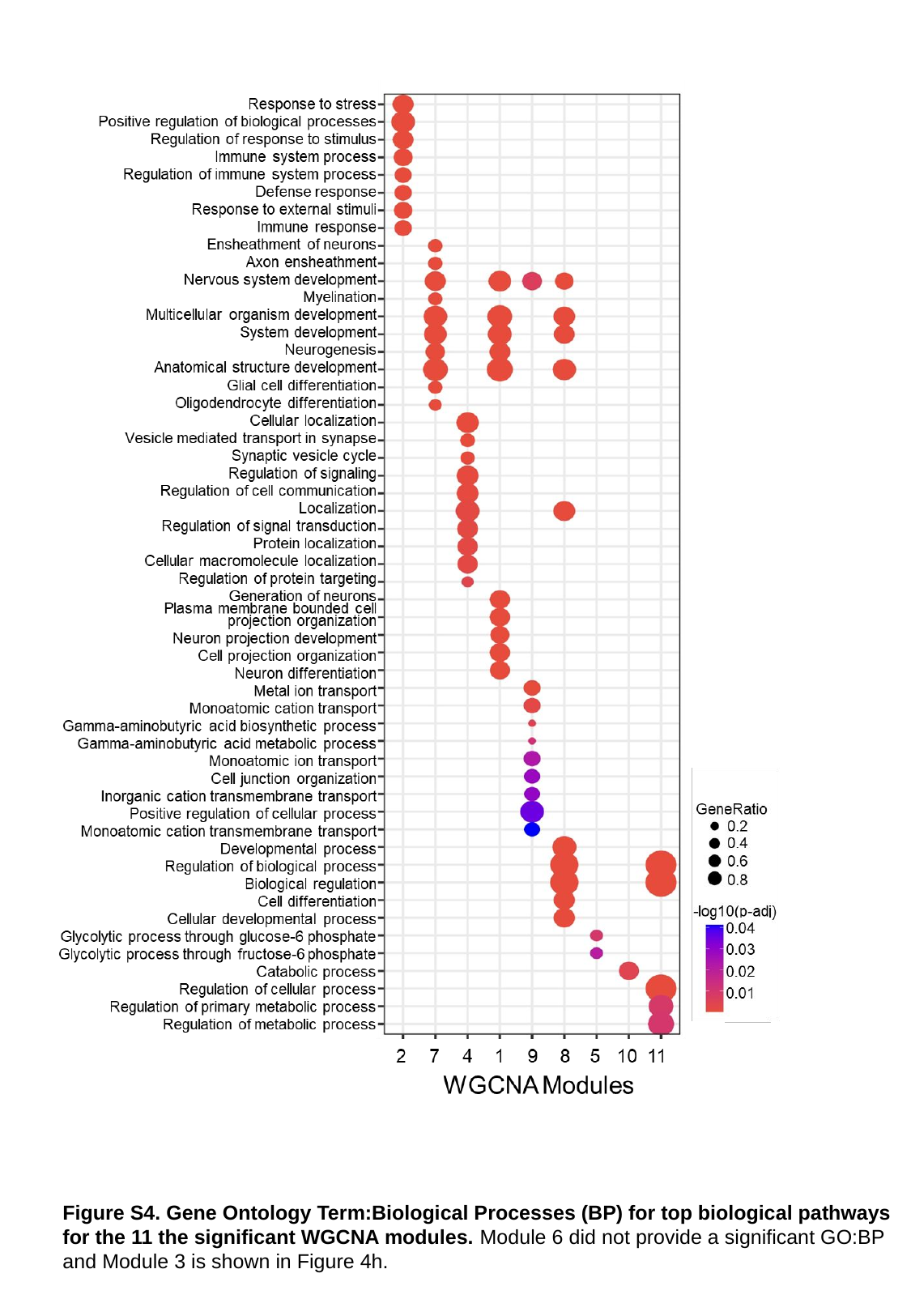

Figure S4. Gene Ontology Term:Biological Processes (BP) for top biological pathways for the 11 the significant WGCNA modules. Module 6 did not provide a significant GO:BP and Module 3 is shown in Figure 4h.

## Slide 7
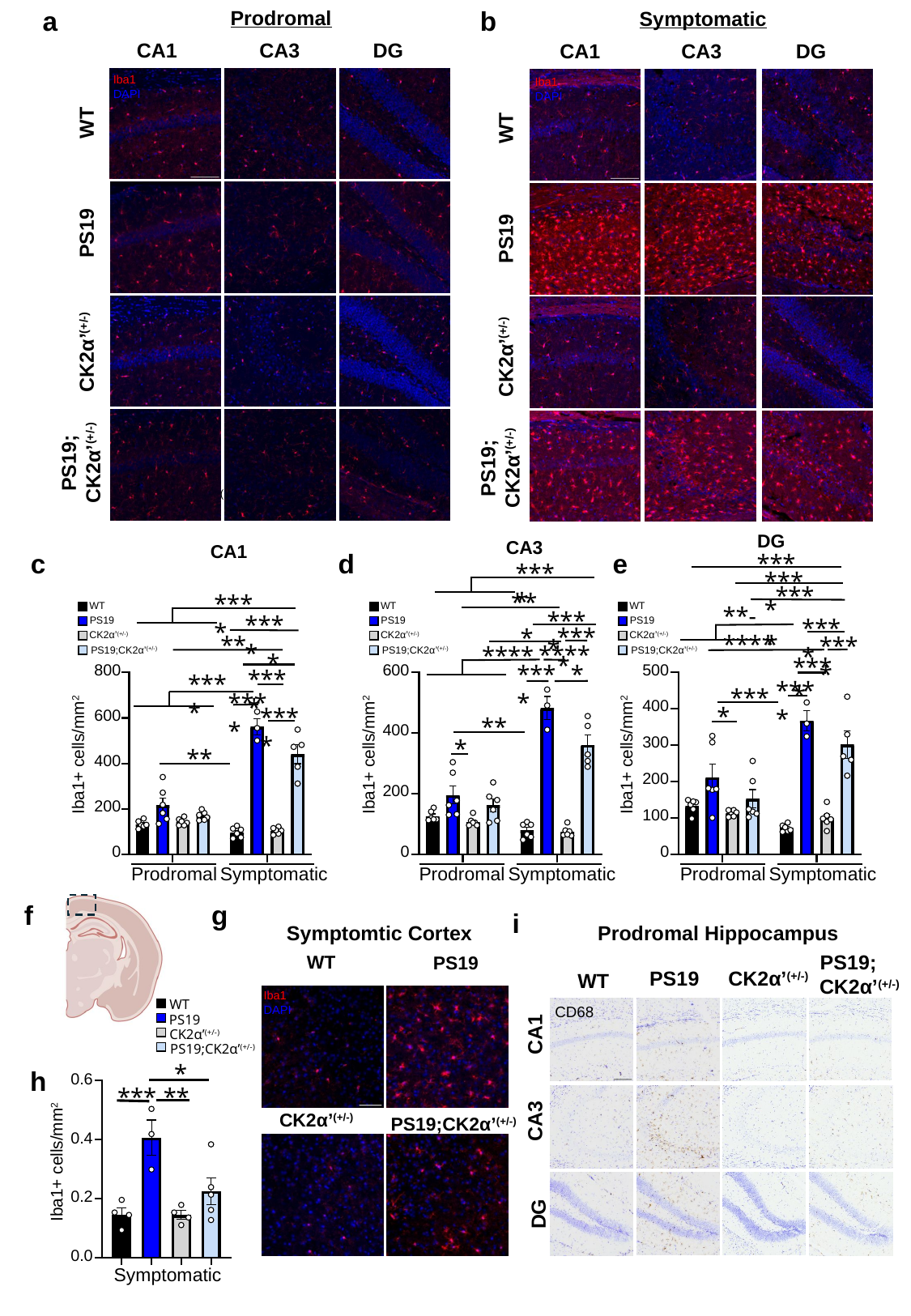

a
b
Prodromal
CA1
CA3
DG
Iba1
DAPI
WT
PS19
CK2α’(+/-)
PS19;
CK2α’(+/-)
CK2α’(+/-)
Symptomatic
CA1
CA3
DG
Iba1
DAPI
WT
PS19
CK2α’(+/-)
PS19;
CK2α’(+/-)
DG
500
400
300
200
100
0
Iba1+ cells/mm2
Prodromal
Symptomatic
CA3
CA1
c
d
e
***
****
****
***
**
****
WT
PS19
CK2α’(+/-)
PS19;CK2α’(+/-)
WT
PS19
CK2α’(+/-)
PS19;CK2α’(+/-)
WT
PS19
CK2α’(+/-)
PS19;CK2α’(+/-)
**-****
****
****
****
****
*
*
**
****
****
****
*
****
WT
PS19
CK2α’(+/-)
PS19;CK2α’(+/-)
600
400
200
0
Iba1+ cells/mm2
Prodromal
Symptomatic
WT
PS19
CK2α’(+/-)
PS19;CK2α’(+/-)
*
800
600
400
200
0
****
****
****
****
***
****
*
****
**
*
**
Iba1+ cells/mm2
Prodromal
Symptomatic
f
g
i
Symptomtic Cortex
WT
PS19
Iba1
DAPI
CK2α’(+/-)
PS19;CK2α’(+/-)
Prodromal Hippocampus
PS19;
 CK2α’(+/-)
PS19
CK2α’(+/-)
WT
CD68
CA1
CA3
DG
WT
PS19
CK2α’(+/-)
PS19;CK2α’(+/-)
h
*
0.6
0.4
0.2
0.0
Iba1+ cells/mm2
Symptomatic
**
***

## Slide 8
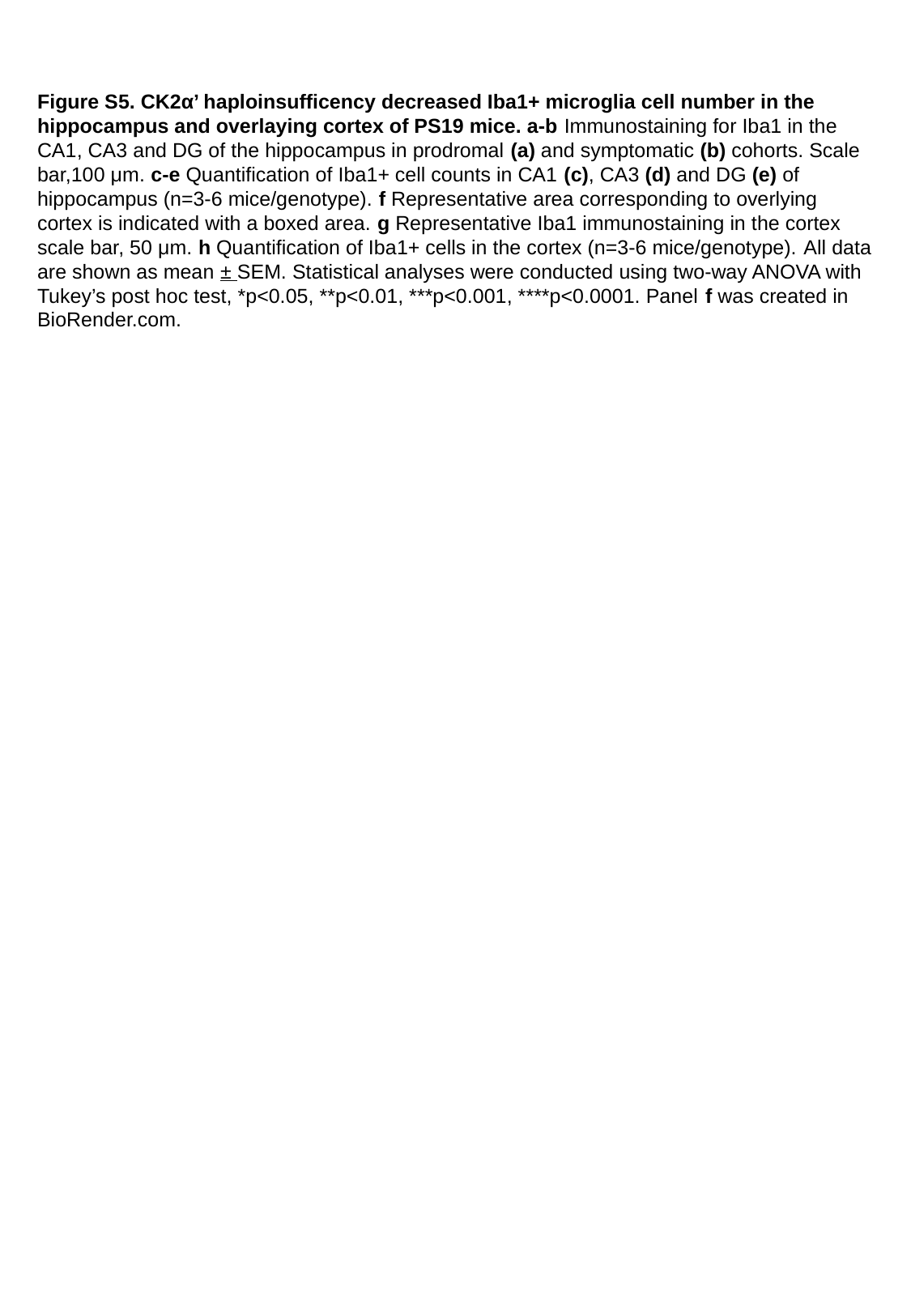

Figure S5. CK2α’ haploinsufficency decreased Iba1+ microglia cell number in the hippocampus and overlaying cortex of PS19 mice. a-b Immunostaining for Iba1 in the CA1, CA3 and DG of the hippocampus in prodromal (a) and symptomatic (b) cohorts. Scale bar,100 μm. c-e Quantification of Iba1+ cell counts in CA1 (c), CA3 (d) and DG (e) of hippocampus (n=3-6 mice/genotype). f Representative area corresponding to overlying cortex is indicated with a boxed area. g Representative Iba1 immunostaining in the cortex scale bar, 50 μm. h Quantification of Iba1+ cells in the cortex (n=3-6 mice/genotype). All data are shown as mean ± SEM. Statistical analyses were conducted using two-way ANOVA with Tukey’s post hoc test, *p<0.05, **p<0.01, ***p<0.001, ****p<0.0001. Panel f was created in BioRender.com.

## Slide 9
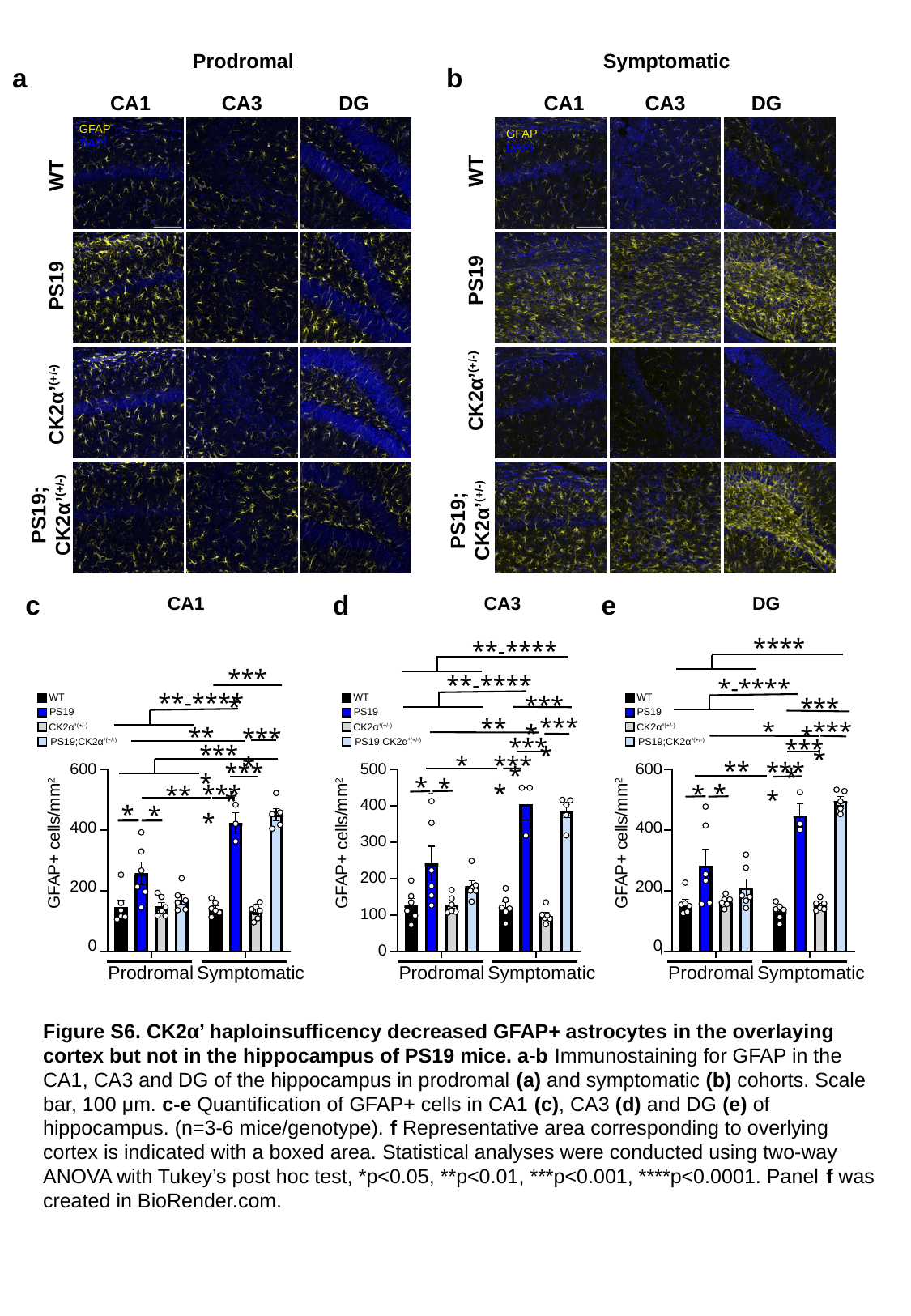

Prodromal
Symptomatic
CA1
CA3
DG
GFAP
DAPI
WT
PS19
CK2α’(+/-)
PS19;
CK2α’(+/-)
a
b
CA1
CA3
DG
GFAP
DAPI
WT
PS19
CK2α’(+/-)
PS19;
CK2α’(+/-)
c
d
e
CA1
CA3
DG
****
**-****
****
**-****
*-****
**-****
****
WT
PS19
CK2α’(+/-)
PS19;CK2α’(+/-)
WT
PS19
CK2α’(+/-)
PS19;CK2α’(+/-)
WT
PS19
CK2α’(+/-)
PS19;CK2α’(+/-)
****
E)
****
**
*
****
**
****
****
****
****
*
****
**
****
****
600
400
200
0
500
400
300
200
100
0
600
400
200
0
*
*
*
****
*
**
b
*
*
GFAP+ cells/mm2
GFAP+ cells/mm2
GFAP+ cells/mm2
Prodromal
Symptomatic
Prodromal
Symptomatic
Prodromal
Symptomatic
Figure S6. CK2α’ haploinsufficency decreased GFAP+ astrocytes in the overlaying cortex but not in the hippocampus of PS19 mice. a-b Immunostaining for GFAP in the CA1, CA3 and DG of the hippocampus in prodromal (a) and symptomatic (b) cohorts. Scale bar, 100 μm. c-e Quantification of GFAP+ cells in CA1 (c), CA3 (d) and DG (e) of hippocampus. (n=3-6 mice/genotype). f Representative area corresponding to overlying cortex is indicated with a boxed area. Statistical analyses were conducted using two-way ANOVA with Tukey’s post hoc test, *p<0.05, **p<0.01, ***p<0.001, ****p<0.0001. Panel f was created in BioRender.com.

## Slide 10
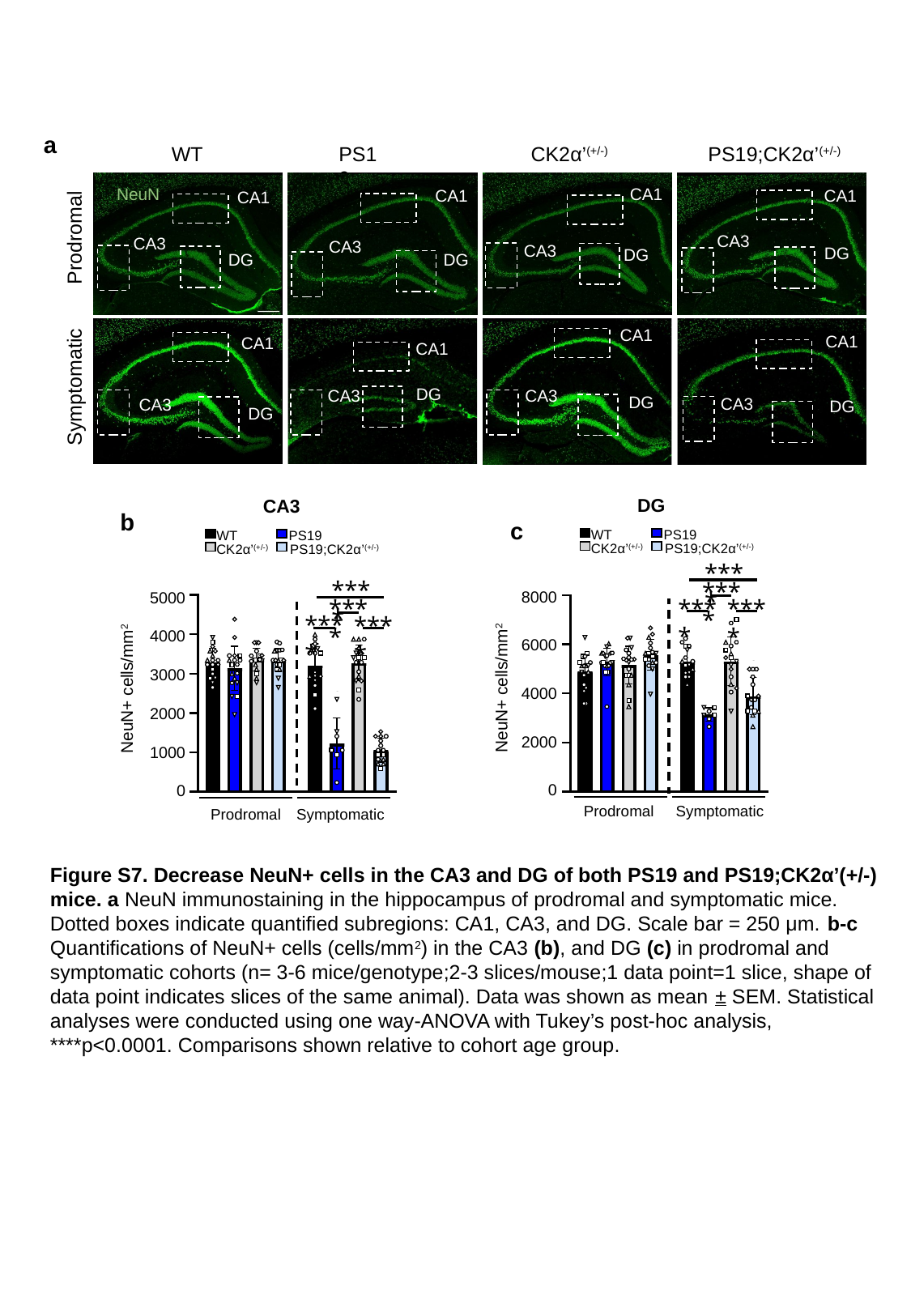

a
PS19
CK2α’(+/-)
PS19;CK2α’(+/-)
WT
CA1
NeuN
CA1
CA1
CA1
Prodromal
CA3
CA3
CA3
CA3
DG
DG
DG
DG
CA1
CA1
CA1
CA1
Symptomatic
DG
CA3
CA3
DG
CA3
CA3
DG
DG
DG
CA3
b
c
WT
PS19
CK2α’(+/-)
PS19;CK2α’(+/-)
WT
PS19
CK2α’(+/-)
PS19;CK2α’(+/-)
****
****
****
8000
6000
4000
2000
0
5000
4000
3000
2000
1000
0
****
****
****
****
****
NeuN+ cells/mm2
NeuN+ cells/mm2
Prodromal
Symptomatic
Prodromal
Symptomatic
Figure S7. Decrease NeuN+ cells in the CA3 and DG of both PS19 and PS19;CK2α’(+/-) mice. a NeuN immunostaining in the hippocampus of prodromal and symptomatic mice. Dotted boxes indicate quantified subregions: CA1, CA3, and DG. Scale bar = 250 μm. b-c Quantifications of NeuN+ cells (cells/mm2) in the CA3 (b), and DG (c) in prodromal and symptomatic cohorts (n= 3-6 mice/genotype;2-3 slices/mouse;1 data point=1 slice, shape of data point indicates slices of the same animal). Data was shown as mean ± SEM. Statistical analyses were conducted using one way-ANOVA with Tukey’s post-hoc analysis, ****p<0.0001. Comparisons shown relative to cohort age group.

## Slide 11
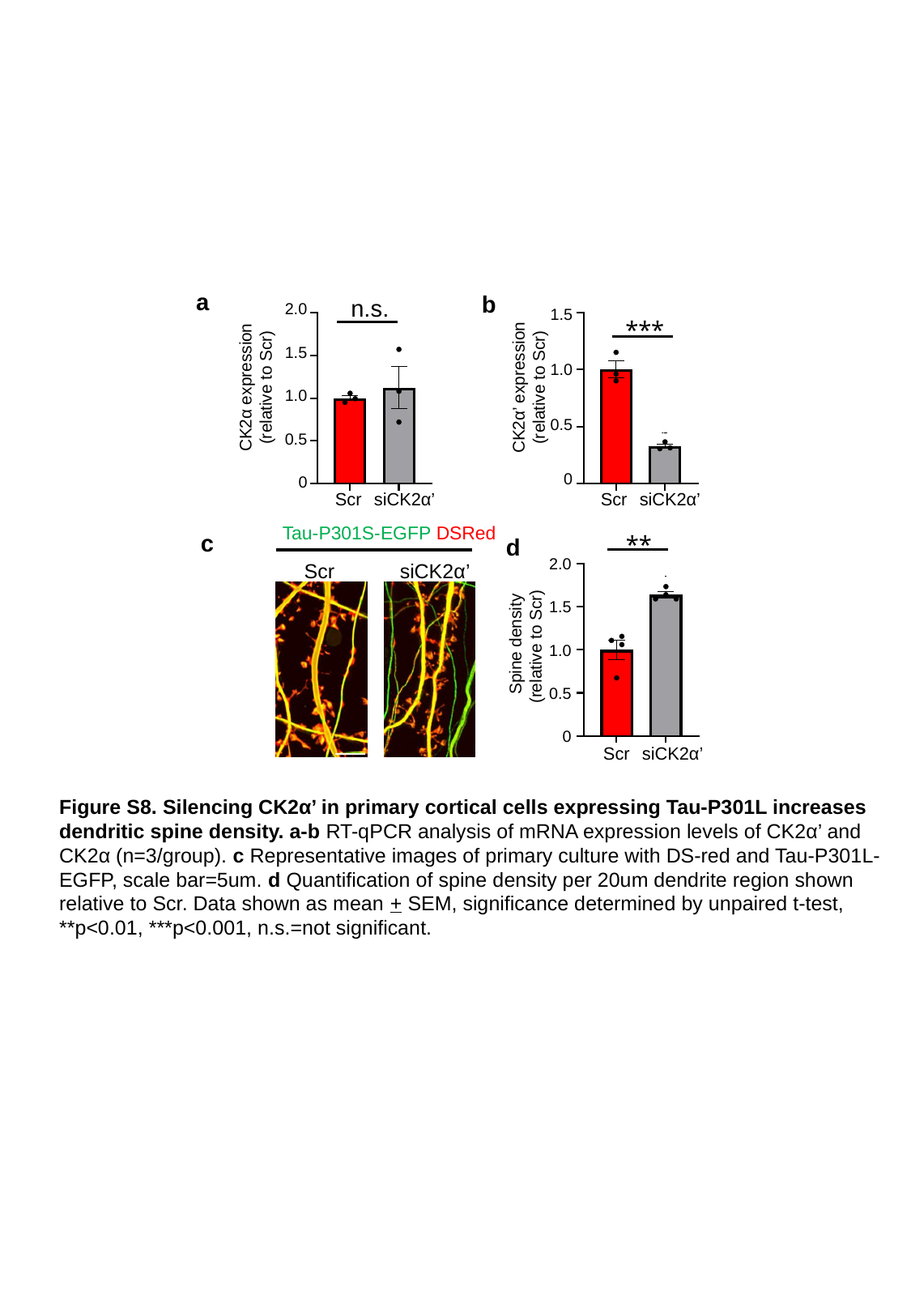

a
b
2.0
1.5
1.5
1.0
CK2α expression
(relative to Scr)
CK2α’ expression
(relative to Scr)
1.0
0.5
0.5
0
0
siCK2α’
Scr
siCK2α’
Scr
Tau-P301S-EGFP DSRed
c
d
2.0
Scr
siCK2α’
1.5
 Spine density
(relative to Scr)
1.0
0.5
0
siCK2α’
Scr
n.s.
***
**
Figure S8. Silencing CK2α’ in primary cortical cells expressing Tau-P301L increases dendritic spine density. a-b RT-qPCR analysis of mRNA expression levels of CK2α’ and CK2α (n=3/group). c Representative images of primary culture with DS-red and Tau-P301L-EGFP, scale bar=5um. d Quantification of spine density per 20um dendrite region shown relative to Scr. Data shown as mean + SEM, significance determined by unpaired t-test, **p<0.01, ***p<0.001, n.s.=not significant.

## Slide 12
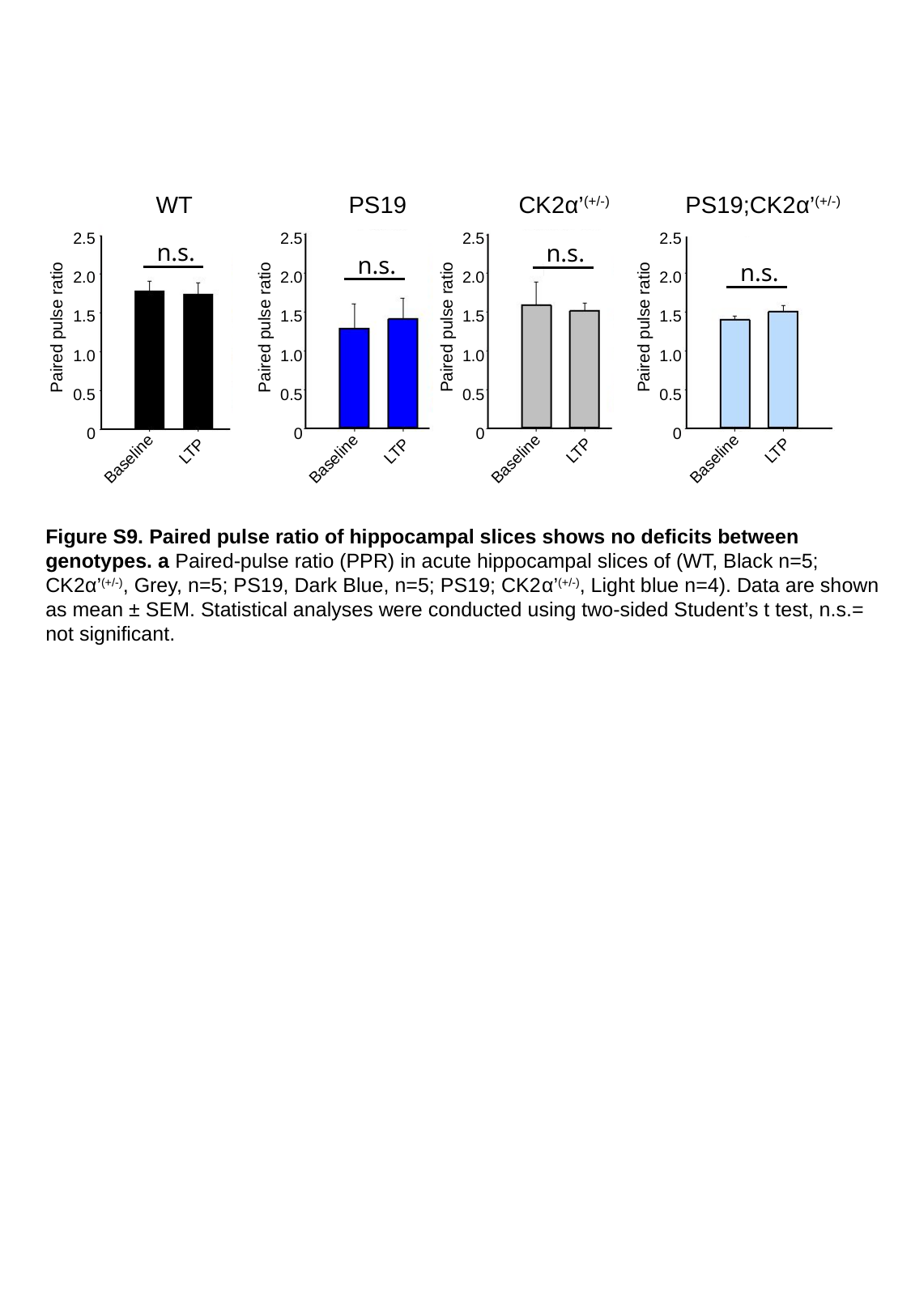

WT
PS19
2.5
2.0
1.5
1.0
0.5
0
Paired pulse ratio
2.5
2.0
1.5
1.0
0.5
0
Paired pulse ratio
LTP
Baseline
LTP
Baseline
CK2α’(+/-)
PS19;CK2α’(+/-)
2.5
2.0
1.5
1.0
0.5
0
Paired pulse ratio
2.5
2.0
1.5
1.0
0.5
0
Paired pulse ratio
LTP
Baseline
LTP
Baseline
n.s.
n.s.
n.s.
n.s.
Figure S9. Paired pulse ratio of hippocampal slices shows no deficits between genotypes. a Paired-pulse ratio (PPR) in acute hippocampal slices of (WT, Black n=5; CK2α’(+/-), Grey, n=5; PS19, Dark Blue, n=5; PS19; CK2α’(+/-), Light blue n=4). Data are shown as mean ± SEM. Statistical analyses were conducted using two-sided Student’s t test, n.s.= not significant.

## Slide 13
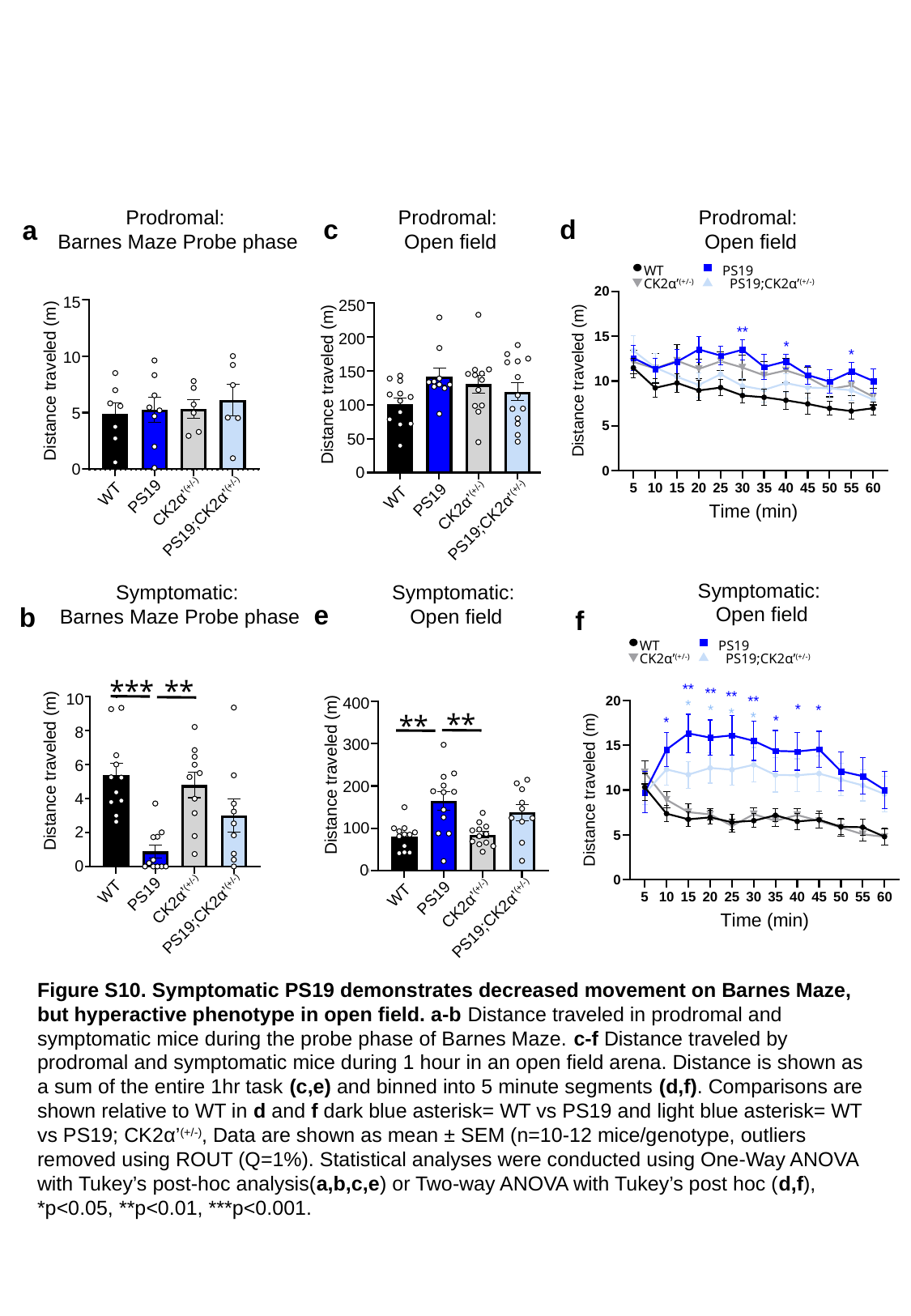

Prodromal:
Barnes Maze Probe phase
Prodromal:
Open field
Prodromal:
Open field
d
c
a
WT
PS19
CK2α’(+/-)
PS19;CK2α’(+/-)
15
10
5
0
Distance traveled (m)
WT
PS19
CK2α’(+/-)
PS19;CK2α’(+/-)
250
200
150
100
50
0
Distance traveled (m)
WT
PS19
CK2α’(+/-)
PS19;CK2α’(+/-)
Symptomatic:
Open field
Symptomatic:
Barnes Maze Probe phase
Symptomatic:
Open field
e
b
f
WT
PS19
CK2α’(+/-)
PS19;CK2α’(+/-)
10
8
6
4
2
0
Distance traveled (m)
WT
PS19
CK2α’(+/-)
PS19;CK2α’(+/-)
***
**
***
**
400
300
200
100
0
Distance traveled (m)
WT
PS19
CK2α’(+/-)
PS19;CK2α’(+/-)
**
**
**
**
Figure S10. Symptomatic PS19 demonstrates decreased movement on Barnes Maze, but hyperactive phenotype in open field. a-b Distance traveled in prodromal and symptomatic mice during the probe phase of Barnes Maze. c-f Distance traveled by prodromal and symptomatic mice during 1 hour in an open field arena. Distance is shown as a sum of the entire 1hr task (c,e) and binned into 5 minute segments (d,f). Comparisons are shown relative to WT in d and f dark blue asterisk= WT vs PS19 and light blue asterisk= WT vs PS19; CK2α’(+/-), Data are shown as mean ± SEM (n=10-12 mice/genotype, outliers removed using ROUT (Q=1%). Statistical analyses were conducted using One-Way ANOVA with Tukey’s post-hoc analysis(a,b,c,e) or Two-way ANOVA with Tukey’s post hoc (d,f), *p<0.05, **p<0.01, ***p<0.001.
