# Supplementary material for: Protein kinase CK2α′ as a dual modulator of neuroimmune signaling and synaptic dysfunction in tauopathy
Source: Transl Neurodegener. 2026 Jul 9;15:31. doi: 10.1186/s40035-026-00563-3 (PMC13348822; doi:10.1186/s40035-026-00563-3)
Supplement: Supplementary file 4 — Additional file 4. Uncropped Blots. [file 40035_2026_563_MOESM4_ESM.pptx]

## Slide 1
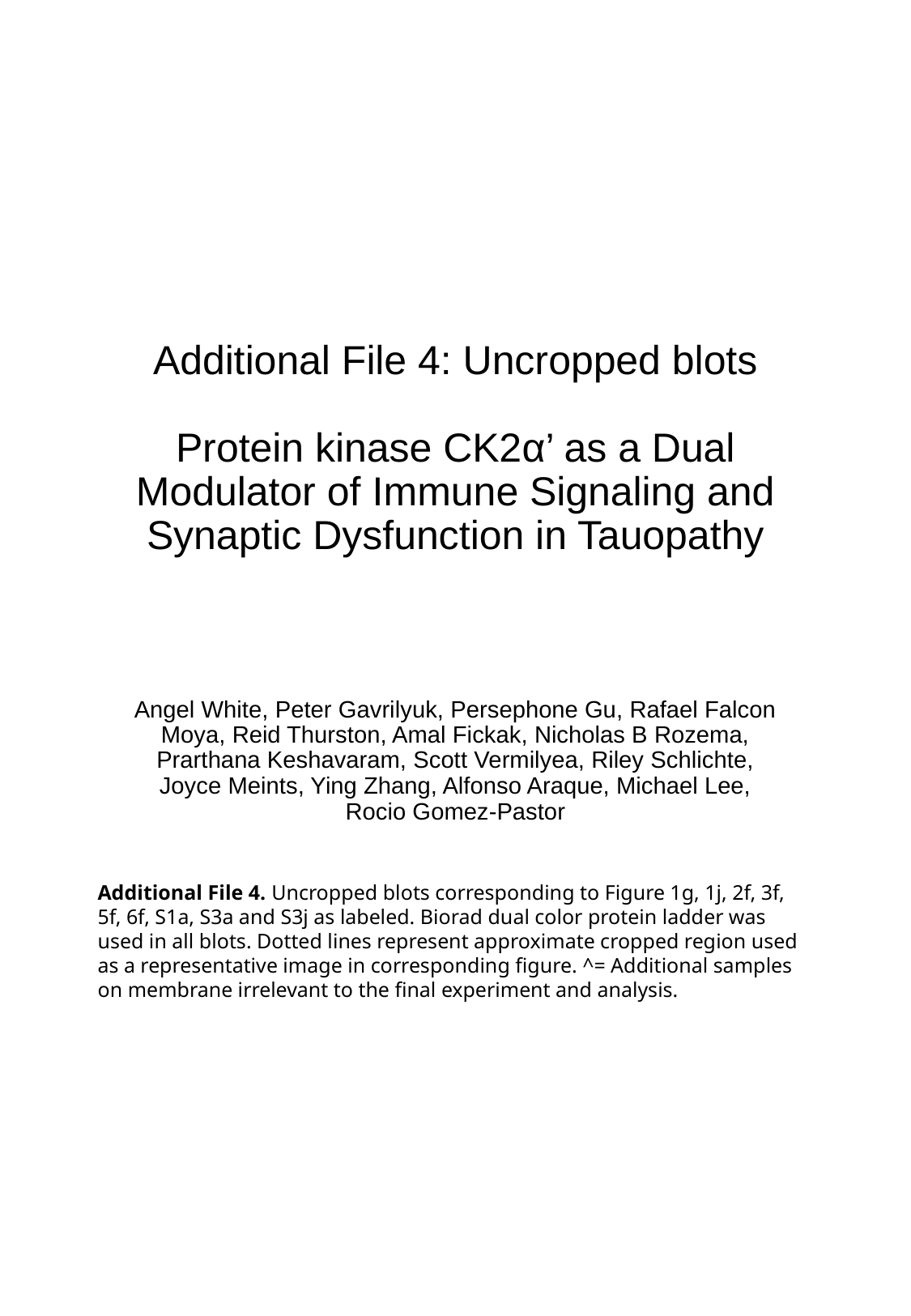

# Additional File 4: Uncropped blots Protein kinase CK2α’ as a Dual Modulator of Immune Signaling and Synaptic Dysfunction in Tauopathy
Angel White, Peter Gavrilyuk, Persephone Gu, Rafael Falcon Moya, Reid Thurston, Amal Fickak, Nicholas B Rozema, Prarthana Keshavaram, Scott Vermilyea, Riley Schlichte, Joyce Meints, Ying Zhang, Alfonso Araque, Michael Lee, Rocio Gomez-Pastor
Additional File 4. Uncropped blots corresponding to Figure 1g, 1j, 2f, 3f, 5f, 6f, S1a, S3a and S3j as labeled. Biorad dual color protein ladder was used in all blots. Dotted lines represent approximate cropped region used as a representative image in corresponding figure. ^= Additional samples on membrane irrelevant to the final experiment and analysis.

## Slide 2
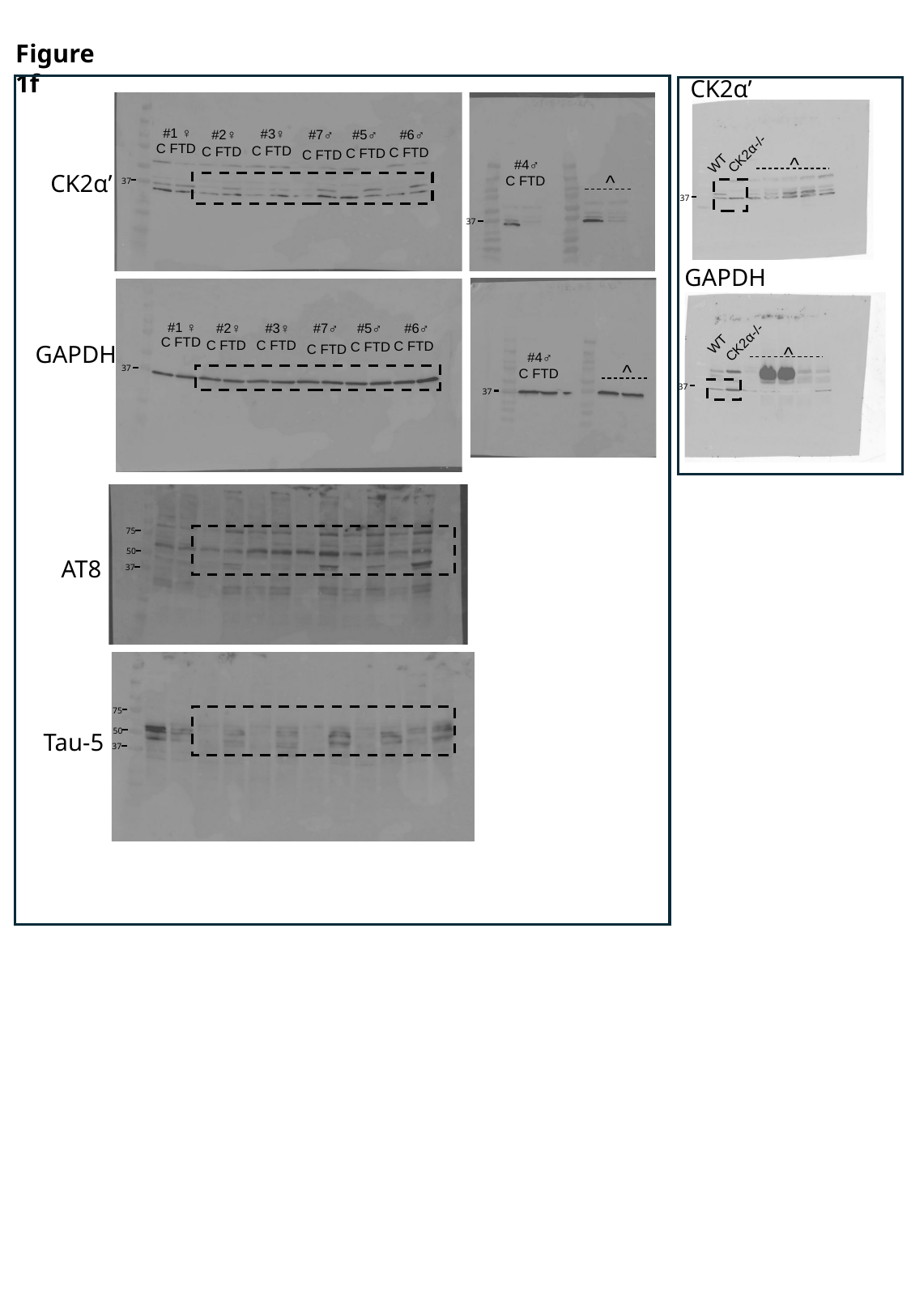

Figure 1f
CK2α’
#1 ♀
#3♀
#2♀
#7♂
#5♂
#6♂
C FTD
C FTD
C FTD
C FTD
C FTD
C FTD
CK2α-/-
WT
^
#4♂
^
CK2α’
C FTD
37
37
37
GAPDH
#1 ♀
#3♀
#2♀
#7♂
#5♂
#6♂
C FTD
C FTD
C FTD
C FTD
C FTD
C FTD
WT
CK2α-/-
^
GAPDH
#4♂
^
37
C FTD
37
37
75
50
AT8
37
75
50
Tau-5
37

## Slide 3
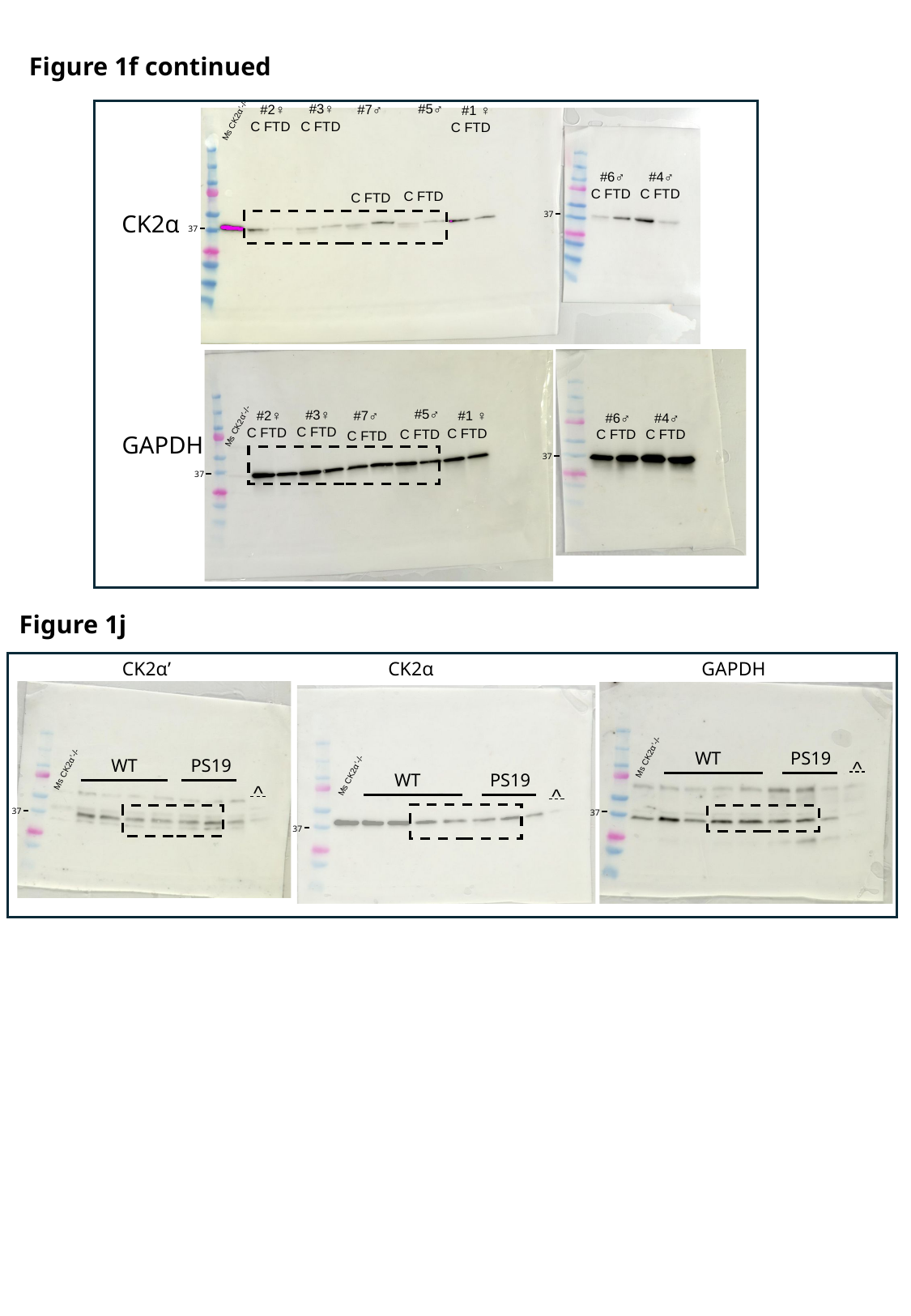

Ms CK2α’-/-
#5♂
#3♀
#2♀
#7♂
#1 ♀
C FTD
C FTD
C FTD
#6♂
#4♂
C FTD
C FTD
C FTD
C FTD
CK2α
Ms CK2α’-/-
#5♂
#3♀
#2♀
#7♂
#1 ♀
C FTD
C FTD
C FTD
#6♂
#4♂
C FTD
C FTD
C FTD
C FTD
GAPDH
Figure 1f continued
37
37
37
37
Figure 1j
CK2α’
CK2α
GAPDH
Ms CK2α’-/-
Ms CK2α’-/-
Ms CK2α’-/-
WT
PS19
^
WT
PS19
WT
PS19
^
^
37
37
37

## Slide 4
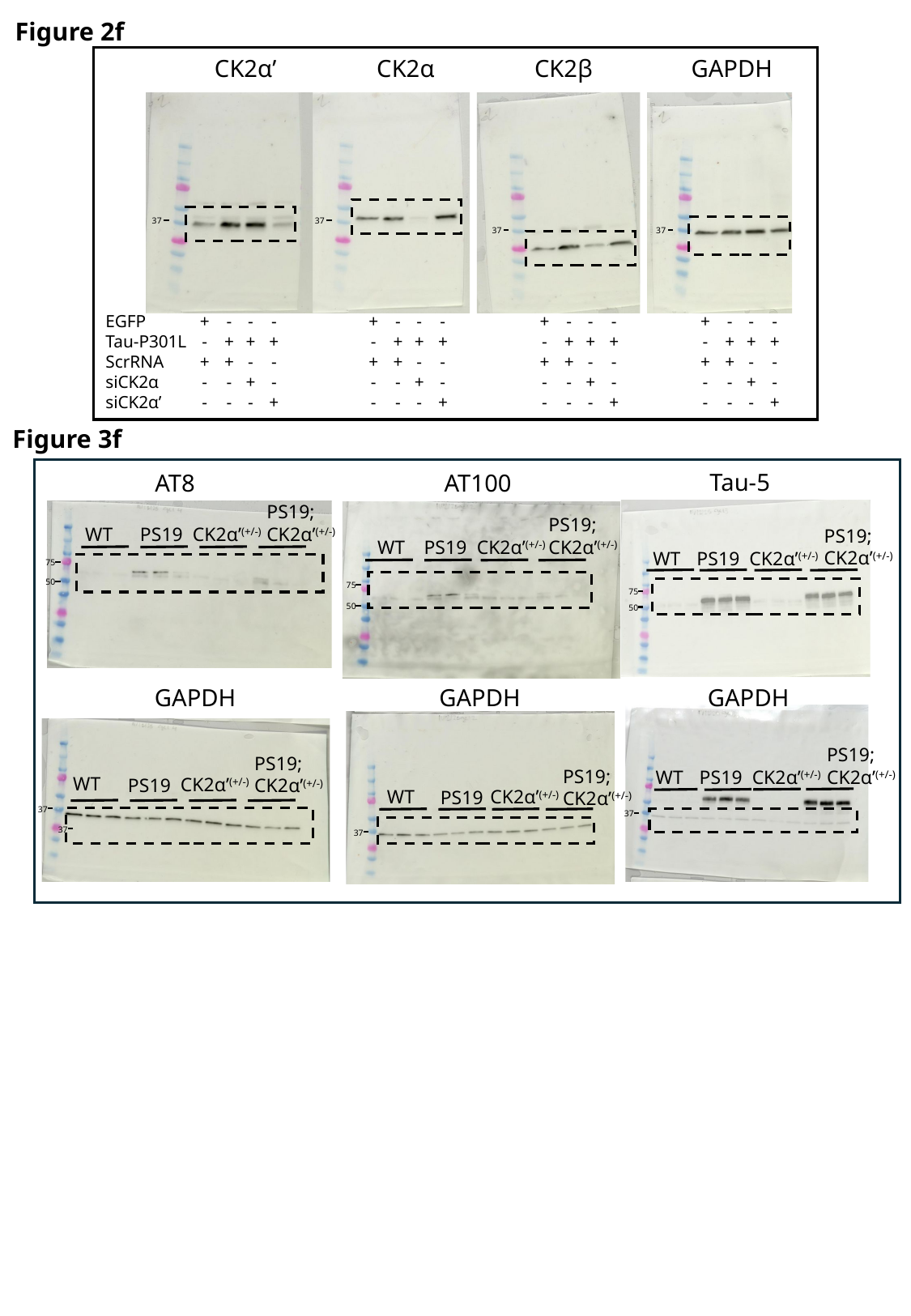

Figure 2f
CK2α’
CK2α
CK2β
GAPDH
EGFP
Tau-P301L
ScrRNA
siCK2α
siCK2α’
+
-
+
-
-
-
+
+
-
-
-
+
-
+
-
-
+
-
-
+
+
-
+
-
-
-
+
+
-
-
-
+
-
+
-
-
+
-
-
+
+
-
+
-
-
-
+
+
-
-
-
+
-
+
-
-
+
-
-
+
+
-
+
-
-
-
+
+
-
-
-
+
-
+
-
-
+
-
-
+
Figure 3f
Tau-5
AT8
AT100
PS19;
CK2α’(+/-)
PS19;
CK2α’(+/-)
WT
PS19
CK2α’(+/-)
PS19;
CK2α’(+/-)
WT
PS19
CK2α’(+/-)
WT
PS19
CK2α’(+/-)
GAPDH
GAPDH
GAPDH
PS19;
CK2α’(+/-)
PS19;
CK2α’(+/-)
PS19;
CK2α’(+/-)
WT
PS19
CK2α’(+/-)
WT
CK2α’(+/-)
PS19
WT
CK2α’(+/-)
PS19
37
37
37
37
75
50
75
75
50
50
37
37
37
37

## Slide 5
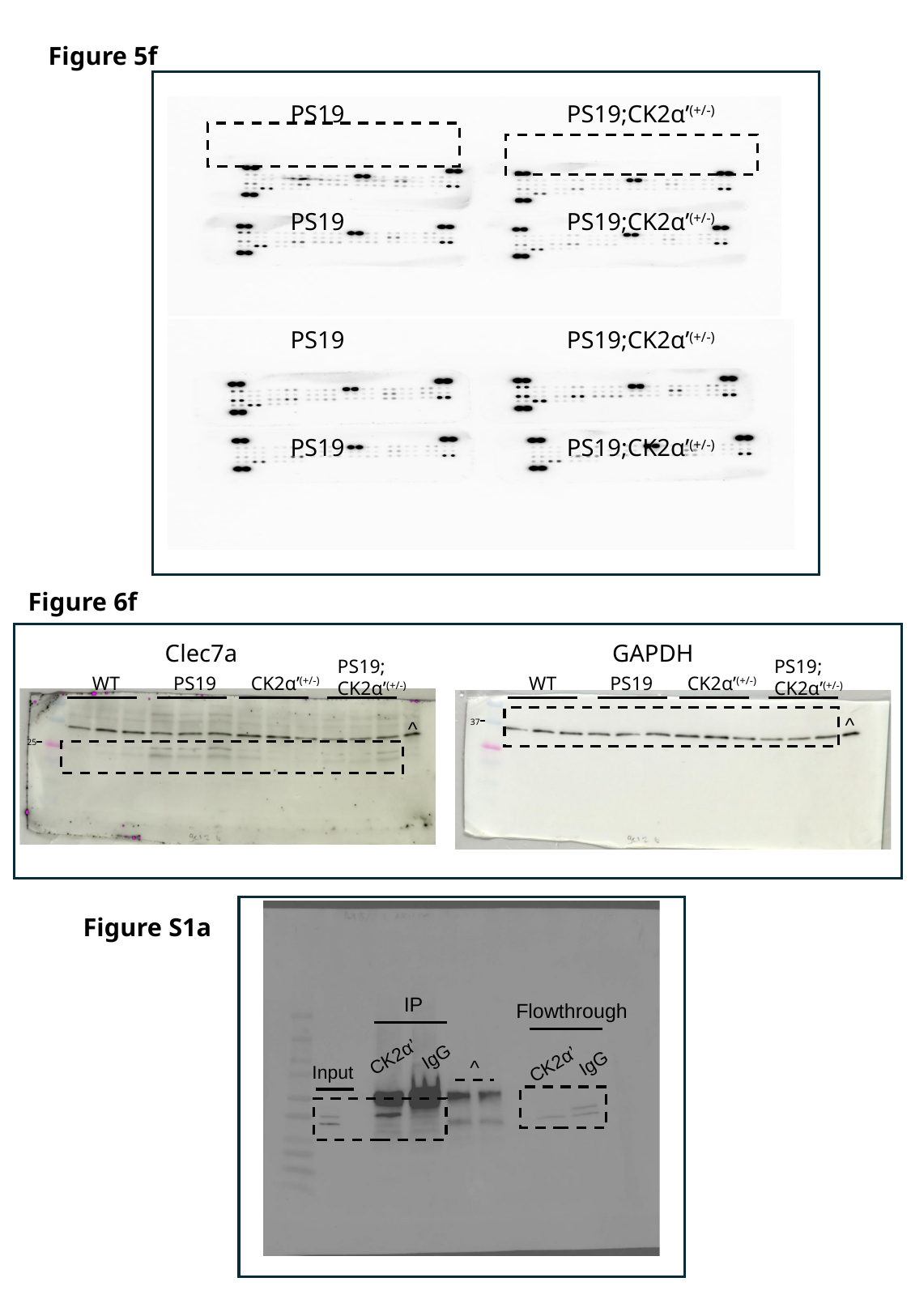

Figure 5f
PS19
PS19;CK2α’(+/-)
PS19
PS19;CK2α’(+/-)
PS19
PS19;CK2α’(+/-)
PS19
PS19;CK2α’(+/-)
Figure 6f
Clec7a
WT
PS19
CK2α’(+/-)
PS19;
CK2α’(+/-)
GAPDH
PS19;
CK2α’(+/-)
WT
PS19
CK2α’(+/-)
^
^
37
25
Figure S1a
IP
Flowthrough
IgG
IgG
CK2α’
CK2α’
^
Input

## Slide 6
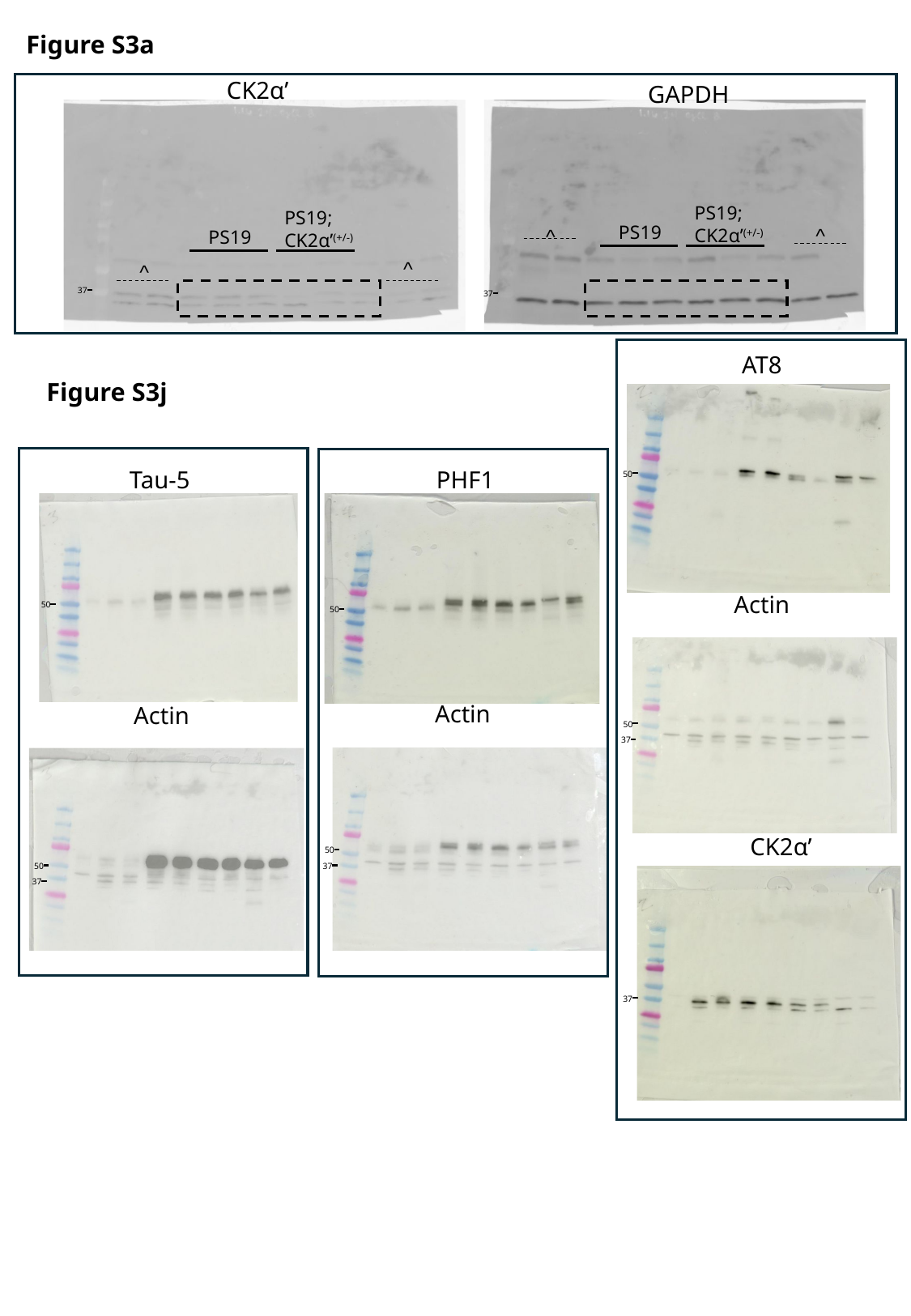

Figure S3a
CK2α’
GAPDH
PS19;
CK2α’(+/-)
PS19
PS19;
CK2α’(+/-)
PS19
^
^
^
^
37
37
AT8
Figure S3j
PHF1
Tau-5
50
Actin
50
50
Actin
Actin
50
37
CK2α’
50
50
37
37
37
